# Causal linkage of presence of mutant NPM1 to efficacy of novel therapeutic agents against AML cells with mutant NPM1

**DOI:** 10.1038/s41375-023-01882-4

**Published:** 2023-03-28

**Authors:** Christopher P. Mill, Warren Fiskus, Kaberi Das, John A. Davis, Christine E. Birdwell, Tapan M. Kadia, Courtney D. DiNardo, Naval Daver, Koichi Takahashi, Koji Sasaki, Gerard M. McGeehan, Xinjia Ruan, Xiaoping Su, Sanam Loghavi, Hagop Kantarjian, Kapil N. Bhalla

**Affiliations:** 1 The University of Texas M.D. Anderson Cancer Center, Houston, TX 77030 USA; 2 Syndax Pharmaceuticals, Waltham, MA USA

**Keywords:** Targeted therapies, Acute myeloid leukaemia

## Abstract

In AML with NPM1 mutation causing cytoplasmic dislocation of NPM1, treatments with Menin inhibitor (MI) and standard AML chemotherapy yield complete remissions. However, the causal and mechanistic linkage of mtNPM1 to the efficacy of these agents has not been definitively established. Utilizing CRISPR-Cas9 editing to knockout (KO) or knock-in a copy of mtNPM1 in AML cells, present studies demonstrate that KO of mtNPM1 from AML cells abrogates sensitivity to MI, selinexor (exportin-1 inhibitor), and cytarabine. Conversely, the knock-in of a copy of mtNPM1 markedly sensitized AML cells to treatment with MI or cytarabine. Following AML therapy, most elderly patients with AML with mtNPM1 and co-mutations in FLT3 suffer AML relapse with poor outcomes, creating a need for novel effective therapies. Utilizing the RNA-Seq signature of CRISPR-edited AML cells with mtNPM1 KO, we interrogated the LINCS1000-CMap data set and found several pan-HDAC inhibitors and a WEE1 tyrosine kinase inhibitor among the top expression mimickers (EMs). Additionally, treatment with adavosertib (WEE1 inhibitor) or panobinostat (pan-HDAC inhibitor) exhibited synergistic in vitro lethal activity with MI against AML cells with mtNPM1. Treatment with adavosertib or panobinostat also reduced AML burden and improved survival in AML xenograft models sensitive or resistant to MI.

## Introduction

Cytoplasmic dislocation and loss of nucleolar localization of the chaperone protein NPM1 occurs in 30% of AML, most commonly following a heterozygous, frame shift, C-terminal mutation in exon 12 of NPM1 (creating NPM1c) [1–3]. This contributes to differentiation arrest, cell cycle growth, and self-renewal of AML stem progenitor cells harboring mutant (mt) NPM1 [4–7]. Patients with AML expressing mtNPM1 display favorable prognosis, exhibiting high complete remission (CR) rates following standard induction and consolidation chemotherapy, or following therapy with hypomethylating agent and venetoclax [8–11]. However, most elderly patients, including those with co-mutation in FLT3, relapse and have a poor clinical outcome [9–12]. Novel treatments that have shown activity against AML with mtNPM1 include arsenic trioxide, which causes degradation of NPM1c, or selinexor the inhibitor of exportin-1 (gene product of XPO1 gene) which blocks cytoplasmic dislocation of NPM1c [13, 14]. However, these therapies have not significantly impacted the overall clinical outcome in AML with mtNPM1. Although stem cell transplantation (SCT) is highly effective and prolongs survival in some patients, its application is limited in elderly patients, and its efficacy mostly observed in MRD (minimal residual disease) negative AML [15–17]. Although lacking in MLL rearrangement (MLLr), AML with mtNPM1 exhibit upregulation of genes involved in stem cell maintenance, including HOXA9 and its co-factors MEIS1 and PBX3, as well as their targets [3, 6, 7, 18]. Most NPM1c is cytoplasmic, but approximately 10% of NPM1c, complexed with exportin-1 protein, binds to a sub-set of gene-enhancers/promoters regulated by Menin-MLL1 and specifically upregulates their mRNA expressions, including those of the leukemogenic HOXA9/MEIS1 [19]. Consistent with this, treatment with Menin inhibitor (MI), e.g., SNDX-50469, SNDX-5613 (revumenib) or ziftomenib, inhibits HOXA9/MEIS1 and induces in vitro growth inhibition, differentiation, and loss of viability of AML cells expressing mtNPM1 [20–23]. Pre-clinically, treatment with MI such as SNDX-5613 or its combinations with venetoclax or FLT3 inhibitors has been shown to exert significant in vivo activity in PDX models of AML with mtNPM1 with or without FLT3 mutation [22, 24, 25]. In early clinical trials, treatment with SNDX-5613 induced complete remissions (CR or CR with partial hematologic recovery) in approximately 21% of heavily pre-treated AML patients with mtNPM1 [26, 27]. However, most patients either failed to respond or relapsed, with an overall survival of 7 months [26, 27]. Clinical trials of combinations of MI with HMA (DNA hypomethylating agent) and venetoclax, or with FLT3 inhibitor, are currently being developed and implemented. Taken together, these observations clearly underscore the need to identify novel agents which would show synergistic activity with MI not only against MI-sensitive mtNPM1 AML but also exhibit promising anti-AML efficacy against mtNPM1 AMLs that demonstrate resistance to MI treatment.

In present studies, we first determined the mechanistic linkage of the presence/absence of mtNPM1 to the active epigenome/transcriptome, protein expression, as well as to cell growth, differentiation, and viability of AML cells. Additionally, we interrogated the causal linkage of the presence/absence of mtNPM1 on therapy responsiveness to standard anti-AML agents, including cytarabine and daunorubicin, as well as to novel agents, including MI, exportin-1 inhibitor (KPT-330) [13], arsenic trioxide or ATRA (all-trans-retinoic acid) [28]. For this, we utilized OCI-AML3 cells that harbor heterozygous mutation in NPM1 and express NPM1c as well as their counterparts from which mtNPM1 had been knocked out (KO) utilizing CRISPR-Cas9 editing. Separately, in OCI-AML2 cells that harbor two wild-type copies of NPM1, a copy of mtNPM1 was knocked-in via CRISPR-Cas9 (OCI-AML2 NPM1^mtA/wt^). Findings presented here demonstrate that, compared to control OCI-AML3, CRISPR-edited OCI-AML3 cells with KO of mtNPM1 exhibited profound depletion of NPM1c and suppression of MI-induced loss of viability. Additionally, NPM1c depletion significantly reduced sensitivity of OCI-AML3 cells to apoptosis induced by KPT-330, cytarabine, and daunorubicin. In contrast, OCI-AML2 NPM1^mtA/wt^ cells exhibited increased levels of HOXA9, MEIS1, and c-Myc, and notably these cells were sensitized in vitro and in vivo to MI-induced anti-AML efficacy. Utilizing the RNA-Seq signature of OCI-AML3 cells with CRISPR-edited depletion of NPM1c, we interrogated the LINCS1000-CMap data set of gene expression signatures and determined that the top expression mimickers (EMs) [29, 30]. These included several pan-HDAC inhibitors and a WEE1 tyrosine kinase inhibitor [31, 32]. Additionally, treatment with adavosertib (WEE1 inhibitor, MK-1775) or panobinostat (pan-HDAC inhibitor) exhibited in vitro and in vivo anti-AML efficacy against mtNPM1-expressing AML cell models that were either sensitive or resistant to MI treatment.

## Materials and methods

### Reagents

SNDX-5613, SNDX-50469, ziftomenib (KO-539), ATRA, SY-1425, Cytarabine, Daunorubicin, Selinexor (KPT-330), Entinostat, Panobinostat, and Adavosertib were obtained from MedChem Express (Monmouth Junction, NJ). Cycloheximide was obtained from Santa Cruz Biotechnology, Inc. (Dallas, TX). All compounds were prepared as 10 mM stocks in 100% DMSO and frozen at −80 °C in 5–10 µL aliquots to allow for single use, thus avoiding multiple freeze-thaw cycles that could result in compound decomposition and loss of activity. For in vivo studies, SNDX-5613 was obtained from Syndax Pharmaceuticals under an MTA and reconstituted per the manufacturer’s instructions.

### Cell line authentication

The cell lines utilized in these studies were authenticated in the Characterized Cell Line Core Facility at M.D. Anderson Cancer Center, Houston TX utilizing STR profiling.

### Primary AML blasts

Patient-derived AML cells samples were obtained with informed consent as part of a clinical protocol approved by the Institutional Review Board of The University of Texas, M.D. Anderson Cancer Center. Mononuclear cells were purified by Ficoll Hypaque (Axis Shield, Oslo, Norway) density centrifugation following the manufacturer’s protocol. Mononuclear cells were washed once with sterile 1× PBS then suspended in complete RPMI media containing 20% FBS. Cells were counted to determine the number of cells isolated prior to immuno-magnetic selection. CD34 + AML blast progenitor cells were purified by immuno-magnetic beads conjugated with anti-CD34 antibody following the manufacturer’s protocol (StemCell Technologies, Vancouver, British Columbia) prior to utilization in the cell viability assays, RNA expression, and immunoblot analyses.

### Assessment of percentage non-viable cells

Following designated treatments (72–96 hours), cultured cell lines or patient-derived (PD) AML blast cells, were washed with 1× PBS, stained with TO-PRO-3 iodide (Cat# T3605, Life Technologies, Carlsbad, CA), and analyzed by flow cytometry on a BD Accuri CFlow-6 flow cytometer (BD Biosciences, San Jose, CA).

### Statistical analysis

Significant differences between values obtained in AML cells treated with different experimental conditions compared to untreated control cells were determined using the Student’s *t* test in GraphPad V9. For the in vivo mouse models, a two-tailed, unpaired *t* test was utilized for comparing total bioluminescent flux. For survival analysis, a Kaplan–Meier plot and a Mantel–Cox log-rank test were utilized for comparisons of different cohorts. *P* values of <0.05 were assigned significance.

## Results

### CRISPR KO of mtNPM1 reduced MEIS1, c-Myc, and c-Myb and inhibited growth and viability of AML cells with mtNPM1

**Fig. 1:**
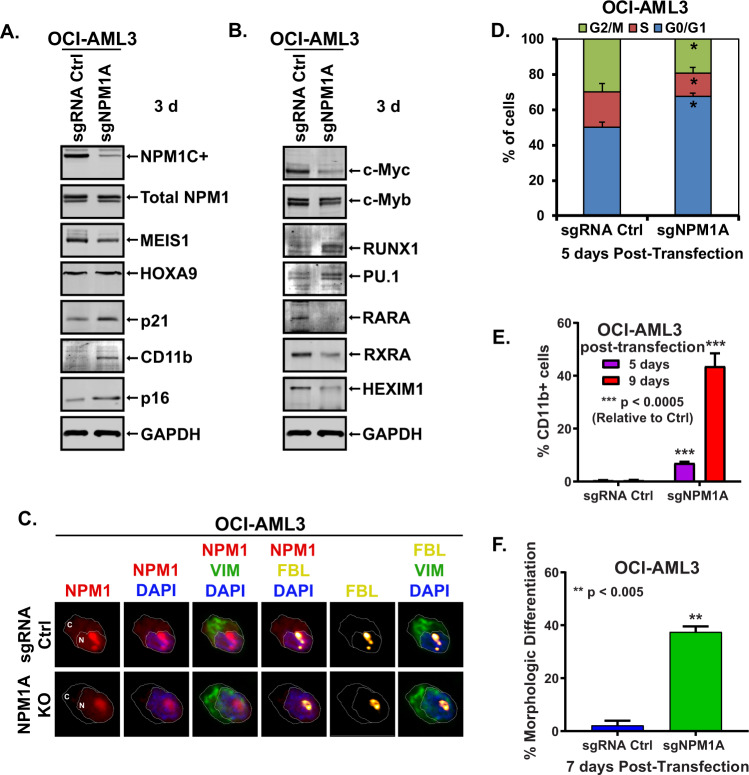
Knockout of mtNPM1 induces cell cycle arrest, depletion of c-Myc, increased expression of p21 and morphologic differentiation of mtNPM1-expressing AML cells. **A**, **B** Immunoblot analyses of OCI-AML3 cells transfected with sgRNA Ctrl or sgNPM1A and incubated for three days. The expression levels of GAPDH were used as the loading control. **C** OCI-AML3 cells were transfected with sgRNA Ctrl or sgNPM1A and incubated for three days. Following this, cells were cytospun onto glass slides, fixed with paraformaldehyde, permeabilized with Triton X-100, and stained with anti-NPM1, Vimentin, and Fibrillarin antibodies. DAPI was used to stain nuclei. Cells were imaged by spinning-disk confocal microscopy. **D** Cell-cycle distribution of OCI-AML3 cells transfected with sgRNA Ctrl or sgNPM1A and incubated for five days. * indicates *p* < 0.05 compared to sgRNA Ctrl. **E** Percent CD11b-positive OCI-AML3 cells transfected with sgRNA Ctrl or sgNPM1A and incubated for five or nine days. **F** Percent differentiated (% myleocytes or metas) OCI-AML3 cells transfected with sgRNA Ctrl or sgNPM1A and incubated for seven days.

Utilizing OCI-AML3 cells that harbor heterozygous mutation in NPM1 and DNMT3A, along with homozygous NRAS mutation [33], we first determined the effects of CRISPR-Cas9 editing of mtNPM1, utilizing specifically designed and targeted gRNA to mtNPM1 [7], on protein expressions of mtNPM1 as well as on AML relevant oncoproteins and on growth and viability of OCI-AML3 cells [34]. Figure 1A and S1A demonstrate that 3 to 8 days following transduction of gRNAs directed at mtNPM1, levels of mtNPM1 and total NPM1 markedly declined. There was concomitant reduction in the protein levels of MEIS1, c-Myc, c-Myb, RARA, and RXRA, but increase in the levels of p21, p16, CD11b, and PU.1 (Fig. 1B and Fig. S1A). Confocal microscopy also demonstrated that following mtNPM1 knockout, there was a profound reduction in cytoplasmic NPM1c and nuclear NPM1 in OCI-AML3 cells, without change in fibrillarin expression in the nucleolus [3] (Fig. 1C). Concomitantly with these perturbations in protein expressions, mtNPM1 KO reduced the % of cells in cell-cycle S phase as well as induced differentiation of OCI-AML3 cells (Figs. 1D–F and S1B). mtNPM1 KO-mediated decline in c-Myc levels was not due to reduced half-life of c-Myc protein, since following cycloheximide co-treatment, c-Myc protein levels declined over 2 hours to a similar extent in OCI-AML3 cells with mtNPM1 KO compared to those treated with the control gRNA (Figs. S1C and S1D). We also determined the effect of CRISPR editing of mtNPM1 in patient-derived (PD) AML cells expressing mtNPM1 (sample #5) (Fig. S1E). As shown in Fig. S1F, the cells transfected with Cas9 and gRNA specific to mtNPM1 induced differentiation associated CD11b and CD14 expressions. Transfection of the gRNA specific to mtNPM1 did not induce the differentiation markers in AML cells harboring wtNPM1 (sample #8) (Figs. S1E and S1G). The mutation profile of the PD AML samples is shown in Fig. S1E

### CRISPR editing of mtNPM1 inhibits enhancers, mRNA gene-sets, mRNA targets of HOXA9/MEIS1, c-Myc and c-Myb, and core regulatory circuitry (CRC)

**Fig. 2:**
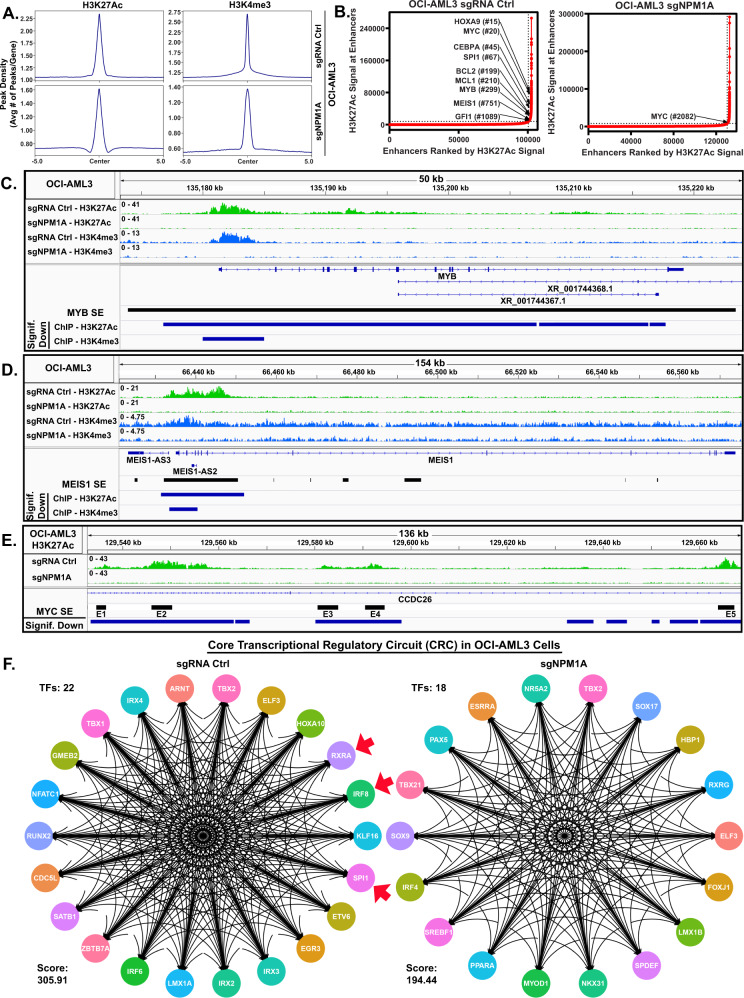
mtNPM1 knockout results in depletion of H3K27Ac and H3K4me3 occupancy at myeloid-relevant enhancers in AML cells. **A** Global H3K27Ac and H3K4me3 peak densities in OCI-AML3 cells transfected with sgRNA Ctrl or sgNPM1A and incubated for five days. **B** ROSE analysis of H3K27Ac ChIP-Seq in OCI-AML3 transfected with sgRNA Ctrl or sgNPM1A and incubated for five days. **C**–**E** IGV plots of H3K27Ac and H3K4me3 signal density at the MYB, MEIS1, and MYC super-enhancer loci in OCI-AML3 cells transfected as in **A**. **F** Knockout of mtNPM1 alters the core transcriptional regulatory circuit (CRC) in OCI-AML3 cells. OCI-AML3 cells transfected with sgRNA Ctrl or sgNPM1A were incubated for five days. Global H3K27Ac ChIP-Seq analysis was utilized to determine the core transcriptional regulatory circuit (CRC). The CRC score is shown for each condition.

**Fig. 3:**
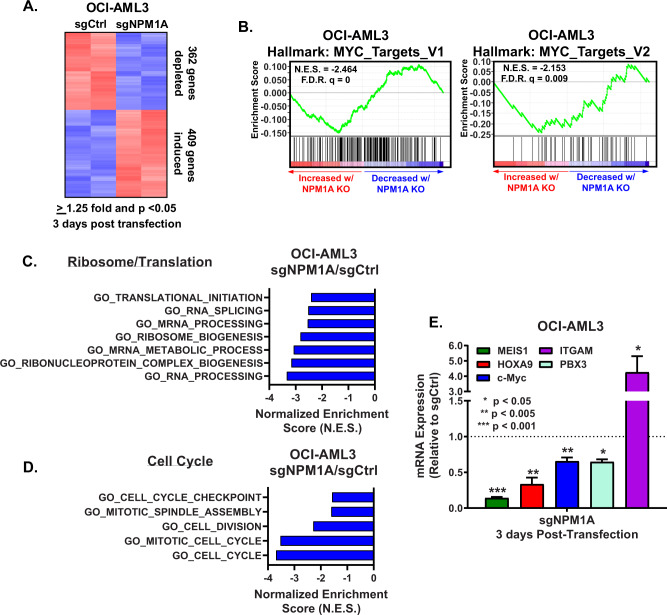
Knockout of mtNPM1 attenuates the ribosome/translation and cell cycle pathways as well as MYC targets in mtNPM1-expressing AML cells. **A** RNA-Seq heatmap of mtNPM1 KO or sgRNA Ctrl in OCI-AML3 cells three days post-transfection. (Biological Replicates) **B**–**D** Gene set enrichment analysis against ribosome/translation, cell cycle (GO pathways), and MYC (HALLMARK) gene sets. **E** QPCR analysis of AML-relevant genes in OCI-AML3 cells with mtNPM1 KO. mRNA expression is normalized to GAPDH and changes in mRNA are relative to sgRNA Ctrl.

To determine the effects of mtNPM1 KO on the active enhancers and promoters in OCI-AML3, we conducted ChIP-Seq analysis to determine peak densities of H3K27Ac and H3K4Me3 at gene-enhancers and/or promoters [35]. Figure 2A demonstrates that 5 days after transduction of the gRNAs directed against mtNPM1, compared to control, there was a significant (*p* < 0.05), log2 fold-decline in the H3K27Ac peak-density at the enhancers/promoters as well as a log2 fold-decline in the H3K4Me3 peak-density at promoters of genes in OCI-AML3 cells. Rank ordering of super-enhancers (SEs) according to their H3K27Ac peak occupancy (ROSE plot) revealed a decline in H3K27Ac mark and associated SE activities of important AML-relevant oncogenes, including HOXA9, MEIS1, MYC, MYB, BCL2, MCL1, GFI1 and SPI1 (Fig. 2B) [36, 37]. Further evaluation of the peak-density plots showed that mtNPM1 KO significantly reduced H3K27Ac and H3K4Me3 occupancy at the MYB SE and promoter, as well as at the promoter of the MEIS1 gene (Fig. 2C, D). H3K27Ac peak-density was also markedly reduced at the 5 enhancers within the SE of MYC present 1.8 Mb downstream of the MYC gene (Fig. 2E) [38]. Peak-density plots of H3K27Ac and H3K4Me3 occupancy showed significantly reduced peak densities at the enhancers and promoters of HOXA and HOXB cluster of genes in OCI-AML3 cells with mtNPM1 KO compared to sgRNA control transfected OCI-AML3 cells (Figs. S2A and S2B). Utilizing H3K27Ac ChIP-Seq data, super enhancer maps, and the core regulatory circuitry (CRC) Mapper algorithm, we determined and compared the CRC in OCI-AML3 mtNPM1 KO versus the control sgRNA-transfected cells [39, 40]. A core transcriptional regulatory circuit (CRC) is a group of interconnected auto-regulating transcription factors (TFs) that form loops and can be identified by super-enhancers (SEs) [39]. As shown in Fig. 2F, compared to the sgRNA control OCI-AML3, sgRNA-mediated KO of mtNPM1 in OCI-AML3 cells attenuated the intensity of CRC and its score from 305 to 194 [39]. Notably, mtNPM1 KO caused the loss of RXRA, IRF8, and SPI1 TFs and their target gene-expressions from the CRC (Fig. 2F). We next determined, via RNA-Seq analysis, the transcriptional impact of mtNPM1 KO due to alterations in the active chromatin in OCI-AML3 cells. Figure 3A demonstrates that 3 days post-transfection of sgRNA directed against mtNPM1, compared to control, mRNA expressions of 409 genes were upregulated, whereas of 362 genes declined (≥1.25-fold and p < 0.05). Among these, gene-expressions of targets of MEIS1/HOXA9, E2F and MYC especially showed negative enrichment scores (Fig. 3B, S3A, S3B). Among the HOXA9/MEIS1 targets, mtNPM1 KO caused a log2 fold-decline in mRNA of HOXB7, FBXO32, SOX4, PIK3R1 and CDKN2C (Fig. S3C). Since both wtNPM1 and c-Myc regulate ribosomal biogenesis, protein translation and cell cycle progression [3, 41, 42], following mtNPM1 depletion by mtNPM1-directed gRNA compared to sgRNA control, the gene-sets involved in protein translation initiation, mRNA splicing/processing and ribosome biogenesis, as well as of cell-cycle checkpoint, mitotic cell cycle and cell division were all negatively enriched (Figs. 3C, D). QPCR analysis confirmed that gRNA-mediated mtNPM1 depletion significantly reduced mRNA expression of HOXA9, MEIS1, PBX3, and MYC, while increasing expression of ITGAM gene (Fig. 3E).

### Effects of CRISPR-mediated depletion of mtNPM1 on activity of retinoids in AML

**Fig. 4:**
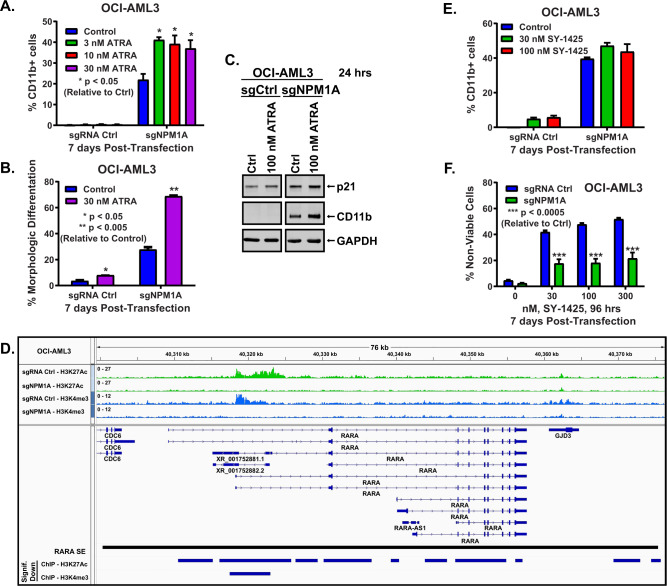
Knockout of mtNPM1 sensitizes OCI-AML3 cells to ATRA-induced differentiation. **A**, **B** Percent CD11b-positive and differentiated (myelocytes and metas) OCI-AML3 cells following three days of KO of mtNPM1 and then treatment with the indicated concentrations of ATRA for 96 h. **C** Immunoblot analyses of OCI-AML3 cells transfected with sgRNA Ctrl or sgNPM1A for 96 hours and then treated with 100 nM of ATRA for 24 hours. **D** IGV plot of H3K27Ac and H3K4me3 signal density at the RARA locus in OCI-AML3 cells transfected with sgRNA Ctrl or sgNPM1A and incubated for 5 days. **E** Percent CD11b-positive differentiated OCI-AML3 cells following three days of KO of mtNPM1 and then treatment with the indicated concentrations of SY-1425 for 96 h. **F** Percent non-viable OCI-AML3 cells following three days of KO of mtNPM1 and then treatment with the indicated concentrations of SY-1425 for 96 h.

Since CRISPR-mediated depletion of mtNPM1 downregulated RXRA and RARA protein levels in OCI-AML3 cells, we tested the effects of treatment with all-trans-retinoic acid (ATRA) in these cells. As shown in Fig. 4A, B, treatment with ATRA-induced CD11b expression and morphologic features of differentiation (% increase in myelocytes and metamyelocytes) in the control OCI-AML3 cells. Notably, CRISPR-mediated depletion of mtNPM1 significantly increased ATRA-induced differentiation in OCI-AML3 cells (Fig. 4A, B). This was associated with an increase in protein levels of p21 and CD11b (Fig. 4C). A previous report had highlighted that increased activity of RARA enhancer correlated with increased sensitivity of PD AML samples to pure RARA agonist tamibarotene (SY-1425) [43]. In OCI-AML3 cells, CRISPR-mediated depletion of mtNPM1 caused a significant decline in H3K27Ac occupancy at the RARA enhancer and promoter and of H3K4Me3 occupancy at the RARA promoter in OCI-AML3 cells (Fig. 4D). While treatment with SY-1425 only slightly induced CD11b expression or loss of viability in the control OCI-AML3 cells, CRISPR-mediated depletion of mtNPM1 and the resulting decline in active chromatin at RARA enhancers did not significantly affect differentiation but reduced the lethal activity of SY-1425 in OCI-AML3 cells (*p* < 0.0005) (Figs. 4E, F). These findings indicate that CRISPR-mediated depletion of NPM1c, sensitized OCI-AML3 cells to ATRA but not to SY-1425-induced differentiation.

### CRISPR-mediated depletion of mtNPM1 blocks activity of Menin inhibitor, exportin-1 inhibitor, cytarabine, and daunorubicin

**Fig. 5:**
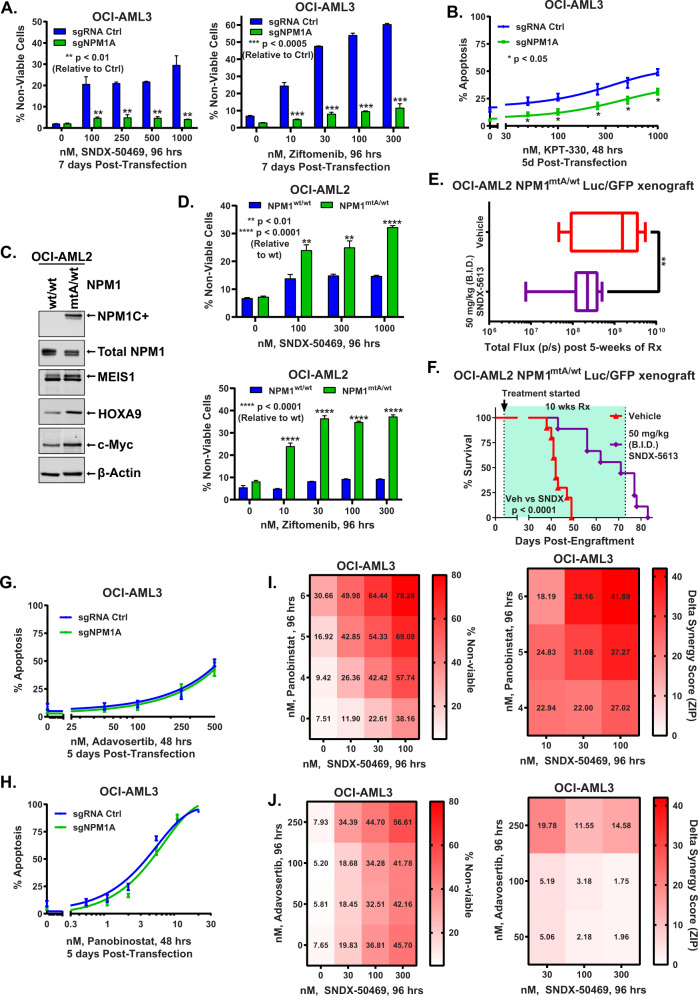
Differential sensitivity mediated by knockout or knock-in of mtNPM1 and synergistic lethal activity of Adavosertib or Panobinostat-based combinations in mtNPM1-expressing AML cells. **A** OCI-AML3 cells with and without mtNPM1 KO were treated with the indicated concentrations of SNDX-50469 or ziftomenib for 96 h. The percent of ToPro-3 iodide-positive, non-viable cells were analyzed by flow cytometry. **B** Percent apoptotic cells following KO of mtNPM1 and treatment with selinexor (KPT-330) at the indicated concentrations for 48 hours. **C** Immunoblot analysis of OCI-AML2 cells with and without CRISPR-mediated knock-in of mtNPM1. **D** Loss of viability in OCI-AML2 cells with and without knock-in of mtNPM1 treated with the indicated concentrations of SNDX-50469 or ziftomenib for 96 h. **E** Total bioluminescent flux (p/s) in NSG mice engrafted with luciferase-expressing OCI-AML2 NPM1^mtA/wt^ cells and treated for five weeks as indicated. ** indicates *p* < 0.01 compared to vehicle. **F** Kaplan–Meier survival curve of NSG mice engrafted with OCI-AML2 NPM1^mtA/wt^ cells and treated with SNDX-5613 for ten weeks. **G**, **H** OCI-AML3 cells transfected with sgRNA Ctrl or sgNPM1A for 72 hours were treated with the indicated concentrations of Adavosertib or Panobinostat for 48 hours. Percent apoptotic cells were determined by flow cytometry. **I**, **J** OCI-AML3 cells were treated with the indicated concentrations of SNDX-50469 and/or Panobinostat or Adavosertib for 96 hours. Percent non-viable cells were determined by flow cytometry. Delta synergy scores were calculated using the ZIP method within the SynergyFinder3.0 web app. Synergy scores >1.0 indicate synergism.

We next determined the mechanistic linkage of the levels of mtNPM1 on the lethal activity of MI, exportin-1 inhibitor, or the conventional DNA-damaging anti-AML drugs, including cytarabine and daunorubicin. As we previously reported, exposure to MI SNDX-50469 or ziftomenib induced loss of viability in OCI-AML3 cells (Fig. 5A) [22, 23]. However, CRISPR-mediated depletion of NPM1c significantly inhibited MI-induced loss of viability in OCI-AML3 cells (Fig. 5A). Compared to sgRNA control, OCI-AML3 cells depleted of NPM1c were also relatively resistant to exportin-1 inhibitor, KPT-330-mediated loss of viability (Fig. 5B). As with these novel therapies, loss of viability induced by conventional anti-AML agents, cytarabine or daunrubicin, was also significantly inhibited in OCI-AML3 cells following CRISPR-mediated depletion of NPM1c (Figs. S4A and S4B). We next developed a human AML cell model, originally with two copies of wtNPM1, into which a copy of mtNPM1 was knocked in. For this, NPM1A mutation (mtA) was knocked in via CRISPR-Cas9 into OCI-AML2 cells with 2 copies of wtNPM1 (OCI-AML2 NPM1^wt/wt^), thus creating the isogenic OCI-AML2 NPM1^mtA/wt^ cells with heterozygous mtNPM1 (Fig. 5C). Immunoblot analysis confirmed that, as compared with the non-edited OCI-AML2 NPM1^wt/wt^ cells, the OCI-AML2 NPM1^mtA/wt^ cells showed expression of NPM1c protein, without significant alteration in the total NPM1 levels (Fig. 5C). Notably, OCI-AML2 NPM1^mtA/wt^ cells displayed higher levels of MEIS1, HOXA9 and c-Myc proteins than OCI-AML2 NPM1^wt/wt^ cells (Fig. 5C). Confocal microscopy confirmed that in OCI-AML2 NPM1^mtA/wt^ cells, NPM1 was cytoplasmic as well as in the nucleolus where it co-localized with fibrillarin, a protein localized to the dense fibrillar core of the phase-separated nucleolar compartment [3] (Fig. S4C). Importantly, treatment with SNDX-50469 or ziftomenib dose-dependently induced significantly greater loss of viability in OCI-AML2 NPM1^mtA/wt^ versus OCI-AML2 NPM1^wt/wt^ cells (Fig. 5D). A low level of sensitivity to MI treatment in OCI-AML2 NPM1^wt/wt^ cells was likely due to a cryptic MLL1r and the presence of MLL1-AF6 fusion protein in these cells [44]. Whereas OCI-AML2 NPM1^wt/wt^ cells were resistant, OCI-AML2 NPM1^mtA/wt^ cells were dose-dependently also more sensitive to cytarabine-induced apoptosis (Fig. S4D). We next tail-vein infused, luciferase-transduced, OCI-AML2 NPM1^mtA/wt^ cells into immune depleted (NSG) mice. Following engraftment, mice were treated via oral gavage with SNDX-5613 versus vehicle control. After 5-weeks of treatment, SNDX-5613-treated mice demonstrated significantly less AML burden (Fig. 5E). Treatment for 10-weeks with SNDX-5613, compared to vehicle control, resulted in significant improvement in survival of the mice without exhibiting any toxicity (Fig. 5F). In contrast, SNDX-5613 treatment of NSG mice engrafted with OCI-AML2 NPM1^wt/wt^ neither significantly reduced the leukemia burden nor improved survival of the mice (Figs. S4E and S4F).

### Inhibitors of WEE1 kinase or histone deacetylases exert in vitro lethal activity against the isogenic AML cells with or without the presence of mtNPM1

**Fig. 6:**
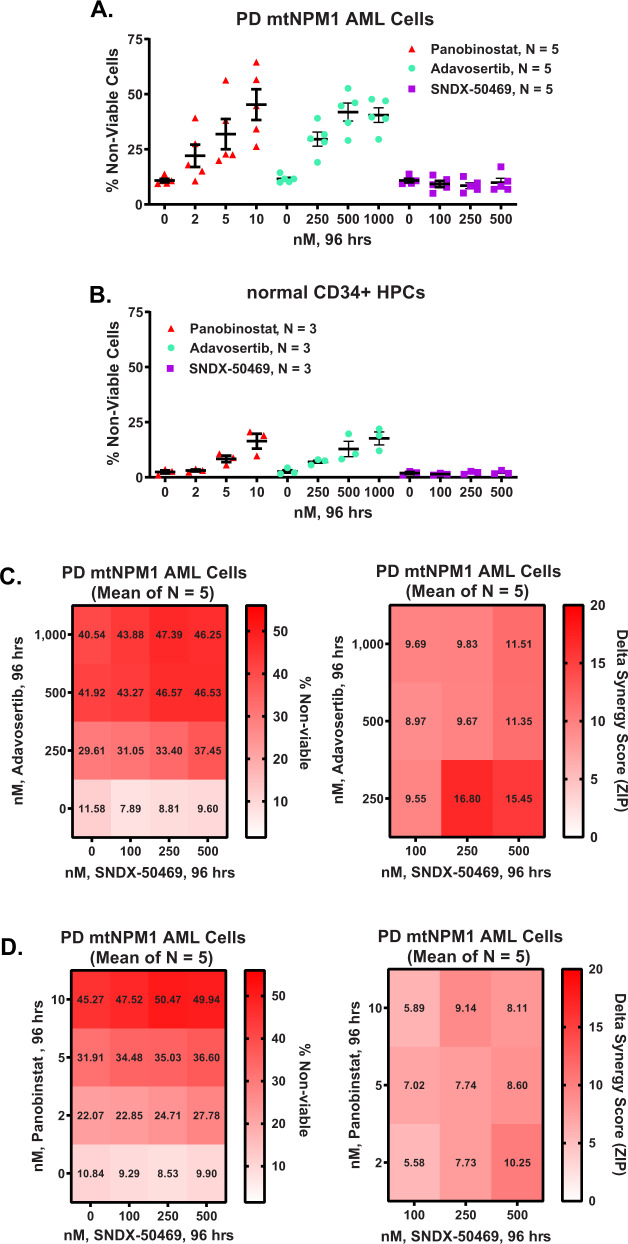
Differential sensitivity of patient-derived (PD) mtNPM1-expressing AML cells to SNDX-50469, Adavosertib, or Panobinostat. **A** PD mtNPM1 AML cells (*N* = 5) were treated with the indicated concentrations of Panobinstat, Adavosertib, or SNDX-50469 for 96 hours. Percent non-viable cells were determined by flow cytometry. **B** Normal CD34 + HPCs (*N* = 3) were treated with the indicated concentrations of Panobinstat, Adavosertib, or SNDX-50469 for 96 hours. Percent non-viable cells were determined by flow cytometry. **C**, **D** PD mtNPM1 AML cells (*N* = 5) were treated with the indicated concentrations of SNDX-50469 and/or Panobinostat or Adavosertib for 96 hours. Percent non-viable cells were determined by flow cytometry. Delta synergy scores were calculated using the ZIP method within the SynergyFinder3.0 web app. Synergy scores >1.0 indicate synergism.

We next used the RNA-Seq signature of mRNA perturbations due to CRISPR KO of mtNPM1 in OCI-AML3 cells to query the LINCS 1000-CMap data sets of >1 million gene expression signatures [29, 30]. A rank-ordered list of the top 25 hits or expression mimickers (EMs) is shown in Table S1. Among the EMs were clinically active HDAC (histone deacetylase) inhibitors, including panobinostat, and the WEE1 kinase inhibitor MK-1775 (adavosertib) [31, 32]. Whereas these agents have been shown to exert anti-AML efficacy in preclinical models of AML, whether this efficacy depends on the presence or absence of mtNPM1 has not been interrogated [31, 32]. Figure 5G, H show that treatment with panobinostat or adavosertib dose-dependently induced apoptosis to a similar extent in OCI-AML3 control and OCI-AML3 cells with KO of mtNPM1, with panobinostat demonstrating more potent lethal activity at low nanomolar concentrations. A separate, clinical grade, HDAC inhibitor entinostat also dose-dependently induced apoptosis that was not significantly different in OCI-AML3 sgRNA control and OCI-AML3 cells with KO of mtNPM1 cells (Fig. S4G) [45]. Via CRISPR-Cas9 and utilizing two separate gRNAs, we also knocked out WEE1 to determine its effect on the viability of OCI-AML3 cells. As shown in Fig. S4H, WEE1 knockout induced 20 to 30% loss of viability in OCI-AML3 cells. We next determined the lethal activity of co-treatment with the MI SNDX-50469 and panobinostat or adavosertib in MI-sensitive OCI-AML3 cells. As shown in Figs. 5I, J, the combination of SNDX-50469 and panobinostat or adavosertib induced synergistic lethality in OCI-AML3 cells, with delta synergy scores by the ZIP method of greater than 5.0. We next determined the lethal activity of adavosertib and panobinostat against PD AML cells expressing mtNPM1. Exposure to adavosertib or panobinostat up to 96 hours dose-dependently induced loss of viability in five samples of PD AML cells that were resistant to SNDX-50469, since the 50% lethal dose of SNDX50469 in these samples was >10 µM (Fig. 6A and Table S2). In contrast, normal CD34+ hematopoietic progenitor cells (HPCs) were relatively insensitive to adavosertib, panobinostat, or SNDX-50469-induced loss of viability (Fig. 6B). Notably, co-treatment with SNDX-50469 and adavosertib or panobinostat synergistically induced loss of viability in PD AML cells with mtNPM1 but not in the normal CD34+ progenitor cells (Fig. 6C, D and S6A, B).

### Adavosertib and panobinostat exert in vivo efficacy in OCI-AML3 xenograft and PDX models of AML with mtNPM1

**Fig. 7:**
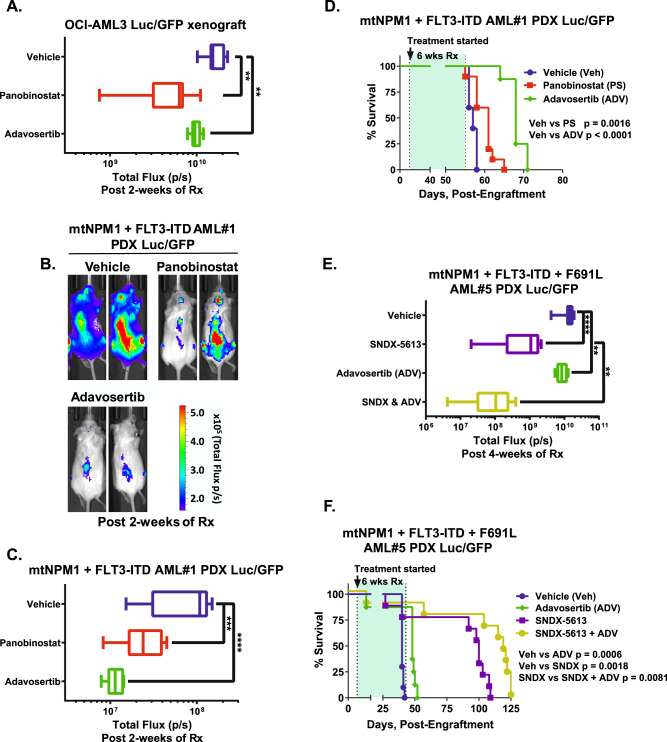
Treatment with Adavosertib or Panobinostat significantly decreases leukemia burden and improves median and overall survival of NSG mice bearing mtNPM1 AML PDX cells. **A** Total bioluminescent flux (p/s) in NSG mice engrafted with (2 million) luciferase-expressing OCI-AML3 cells and treated for two weeks as indicated. ** indicates *p* < 0.01 compared to vehicle. **B** Representative bioluminescent images of leukemia burden from the mice in “A” are shown. **C** Total bioluminescent flux (p/s) in NSG mice engrafted with (~1 million) luciferase-expressing mtNPM1 + FLT3-ITD AML PDX (DF16835) cells and treated for two weeks as indicated. *** indicates *p* < 0.005 and **** indicates *p* < 0.0001 compared to vehicle. **D** Kaplan-Meier survival curve of NSG mice engrafted with mtNPM1 + FLT3-ITD AML PDX cells and treated as indicated for six weeks. **E** Total bioluminescent flux (p/s) in NSG mice engrafted with luciferase-expressing mtNPM1 + FLT3-ITD + F691L AML PDX cells and treated for four weeks as indicated. ** indicates p < 0.01 and **** indicates *p* < 0.0001 compared to vehicle. **F**. Kaplan-Meier survival curve of NSG mice engrafted with mtNPM1 + FLT3-ITD + F691L AML PDX cells and treated as indicated for six weeks. Significance was determined by a Mantel–Cox log-rank test. *P* values less than 0.05 are considered significant.

We next determined the in vivo anti-leukemia efficacy of adavosertib and panobinostat in the OCI-AML3 xenograft model as well as in AML PDX (patient-derived xenograft) models expressing mtNPM1 and FLT3-ITD in NSG mice. AML cells in these models are transduced with Luciferase/GFP for bioluminescence imaging. Following tail-vein infusion and engraftment, cohorts of mice were treated with vehicle control or with panobinostat or adavosertib alone. The doses of each drug employed here were previously determined to be safe [31, 32]. Compared to vehicle control, monotherapy with panobinostat or adavosertib for 2 weeks induced significant reduction in AML burden in the OCI-AML3 xenograft model (Fig. 7A). Additionally, treatment for 2 weeks with the two agents compared to vehicle control also significantly reduced AML burden in an AML PDX mtNPM1-FLT3-ITD model, which also harbored mutations in FLT3-TKD D835Y, PHF6 and ATRX (Figs. 7B, C, and Table S3). Treatment with each drug for 6 weeks also significantly improved the median and overall survival of the NSG mice harboring this PDX model, as compared to treatment with vehicle control (Fig. 7D). Monotherapy with neither panobinostat nor adavosertib was associated with weight loss or other toxicities, as compared to mice treated with vehicle alone. We next evaluated the in vivo efficacy of SNDX-5613 and adavosertib versus each drug alone or vehicle control in NSG mice tail-vein engrafted with a separate and aggressive mtNPM1-FLT3-ITD AML PDX model that also harbored FLT3-F691L TKD. Whereas 4 weeks of treatment with SNDX-5613 significantly reduced AML burden more than adavosertib treatment, co-treatment with SNDX-5613 and adavosertib was significantly superior in reducing the AML burden compared to each drug alone or vehicle control (*p* < 0.05) (Fig. 7E). Co-treatment with SNDX-5613 and adavosertib for 6 weeks was significantly superior to either SNDX-5613 or adavosertib alone in improving median and overall survival of the mice, without inflicting weight loss or other toxicities (Fig. 7F). These in vivo findings indicate that treatment with adavosertib and panobinostat alone or in combination with MI are effective therapies worthy of further in vivo testing and development in AML with mtNPM1 with or without FLT3-ITD.

## Discussion

The presence of NPM1 mutation in AML stem/progenitor cells (LSCs) has been documented to be associated with but not shown to be causally linked to increased sensitivity to AML therapies, including treatment with menin inhibitor, the exportin-1 inhibitor selinexor, RARA or RXR agonists, or with the chemotherapeutic agents cytarabine and daunorubicin. To address this, we created via CRISPR-Cas9 isogeneic AML cell models with depletion (OCI-AML3) or knock-in of mtNPM1 (OCI-AML2). Utilizing these cellular models, we demonstrate that the presence of mtNPM1 is causally linked to the sensitivity of OCI-AML3 cells to loss of viability induced by menin inhibitor, selinexor, or cytarabine.

Compared to a previous report [7], our findings demonstrate a more comprehensive ROSE analysis of the H3K27Ac ChIP-Seq data in OCI-AML3 cells with KO of NPM1c, highlighting the loss of super enhancer at CEBPA, SPI1, BCL2, MCL1, MYB, and GFI1 genes as well as a reduction in the MYC super enhancer ranking. Also, CRISPR-mediated depletion of NPM1c in OCI-AML3 cells markedly perturbed the CRC in AML cells expressing mtNPM1, with significant reduction in the CRC score and abolition of the contribution from the transcriptional regulators PU.1, IRF8, and RXRA to the CRC [7, 39, 40]. Based on the frequency of occurrence of the specific TFs they contain across all the possible interconnected auto regulatory loops in each AML cell sample, the CRCmapper ranked all the possible core regulatory circuitries (CRCs) [39]. The top-scoring circuitry model, containing TFs with the highest frequency of occurrence, was selected as the model of CRC in the control or sgNPM1A transfected OCI-AML3 cells [39]. The CRC score for each sample is calculated by dividing the overall times of occurrence of core TFs across all possible circuitries by the number of core TFs in this circuitry [39, 40]. Loss of RXR, IRF8, and SPI1, and other TFs from the CRC resulted in a lower overall CRC score in OCI-AML3 cells with mtNPM1 KO, compared to the control OCI-AML3 cells. Overall, this highlights that the presence of mtNPM1 sustains the CRC while mtNPM1 KO undermines the CRC in AML. Depletion of NPM1c especially diminished active chromatin at the enhancers and promoters of HOXA and HOXB clusters of genes, as well as of the transcriptional regulators HOXA9, MEIS1, c-Myb, and c-Myc. These findings were associated with the repression of the HOXA9 and its co-factors MEIS1 and PBX3 [46]. In contrast to the report by Brunetti et al, our findings presented here demonstrate that the depletion of NPM1c led to a reduction in the protein levels of important pro-growth and survival factors, including c-Myc and c-Myb [7]. Not shown previously [7], our findings also highlight and explain the negative enrichment of mRNAs of gene-sets belonging to mRNA processing, ribosome biogenesis, protein translation initiation, and cell cycle regulation [42, 47]. NPM1c depletion also increased the levels of p21, p16, CD11b, and PU.1 in OCI-AML3 cells. Collectively, these perturbations due to NPM1c depletion contributed to the induction of cell cycle growth arrest and induction of morphologic features of differentiation in OCI-AML3 cells. Our findings also demonstrate that the depletion of NPM1c levels in OCI-AML3 cells repressed RXRA and reduced RARA enhancer activity. This was associated with decreased protein levels of RXRA and RARA but increased level of differentiation induced by ATRA. A previous report highlighted the link between increased RARA enhancer activity and sensitivity to RARA agonist tamibarotene (SY-1425) [43]. Accordingly, our findings demonstrate that treatment with tamibarotene did not augment differentiation of OCI-AML3 cells with NPM1c depletion. A previous report involving knock-in mouse models of AML had demonstrated that, compared to mice with AML harboring mutant NPM1^cA^ and FLT3-ITD, double mutant NPM1^cA^ and NRAS-G12D model developed AML with a longer latency and exhibited better prognosis [48]. This may explain why in our studies, differentiation induced by NPM1c depletion in OCI-AML3 cells is not prevented by heightened NRAS signaling in OCI-AML3 cells, since OCI-AML3 cells express homozygous Q61L mutation and increased activity of NRAS [33].

Results of preclinical studies and early clinical trials have demonstrated that treatment with MIs, e.g., SNDX-50469, revumenib, and ziftomenib, effectively induce cell differentiation and loss of viability in the preclinical models and induce complete remissions in patients with AML with mtNPM1 with or without FLT3 mutation but not in AML without mtNPM1. Our findings go further and show that KO of mtNPM1 desensitizes AML cells to treatment with MI, selinexor, and cytarabine. Similar to findings presented here that CRISPR-mediated KO of mtNPM1 repressed HOXA genes and MEIS1, in a recent report the targeted degradation of NPM1c or treatment with selinexor of AML cells, was shown to disrupt the binding of the residual and nuclear NPM1c to exportin-1, which led to repression of a small subset of normal MLL1-regulated genes, including HOXA genes and MEIS1, without affecting MLL1 occupancy at the target loci [19]. Additionally, our findings demonstrating that CRISPR-edited knock-in of mtNPM1 led to increased protein levels of HOXA9, MEIS1 and c-Myc, associated with increased in vitro sensitivity and in vivo efficacy to treatment with MI further explains its mechanistic linkage to the presence of mtNPM1.

Given that in most elderly AML patients with mtNPM1, especially with FLT3 co-mutations, MI treatment is either ineffective, or after yielding initial clinical CR, AML relapse and poor clinical outcome ensues. Therefore, we adopted an approach to find agents that would be effective against AML with mtNPM1 regardless of sensitivity/resistance to MI treatment. Utilizing the RNA-Seq signature following NPM1c depletion in OCI-AML3 cells, our findings from LINCS1000-CMap analysis yielded several EMs, including HDAC inhibitors, e.g., THM-I-94, trichostatin-A, apicidin, dacinostat, vorinostat, and panobinostat, as well as adavosertib. Although we elected to fully interrogate the activity of panobinostat, the other FDA-approved pan-HDAC inhibitors, including vorinostat or belinostat, with biologic activity like panobinostat, would be as attractive for further evaluating as monotherapy against AML expressing NPM1c. Nonetheless, findings presented validate that monotherapy with panobinostat or adavosertib exerts in vitro lethal activity and in vivo efficacy against AML expressing NPM1c. Previous reports have documented that, among its diverse mechanisms of activity, as a pan-HDAC inhibitor panobinostat transcriptionally perturbs large numbers of gene-expressions beyond what would be regulated by menin-MLL1 in AML cells, including repression of pro-growth and pro-survival gene-expressions [49]. This would explain its activity in MI-sensitive and MI-resistant AML expressing NPM1c. Similarly, as a WEE kinase inhibitor, adavosertib inhibits cell cycle progression, thus inducing mechanisms of growth inhibition and lethality orthogonal to mechanisms engaged by MI treatment [32]. Collectively, these observations explain why panobinostat, and adavosertib would potentially exert synergistic lethality with MI treatment in AML cells expressing NPM1c.

Recent reports have highlighted the promising preclinical and clinical anti-AML efficacy of other novel agents and their combinations in AML expressing NPM1c [28]. These include novel exportin-1 inhibitors [50, 51], the nucleolar stress-inducing agent dactinomycin [52], the NPM1c protein-degrader arsenic trioxide and ATRA combination [28, 53], and the BH3 mimetic venetoclax based combinations [54]. Immunotherapy of AML expressing mtNPM1 with anti-CD33 or CD123 strategies [55–57], anti-PD1 or PD-L1 antibodies-based combinations [58, 59], or anti-CD123 directed chimeric antigen receptor (CAR-T) cells are also being investigated [60]. However, which of these therapies will be safe and improve clinical outcome in elderly AML expressing mtNPM1 remains to be established. Our findings here have highlighted the novel preclinical efficacy of already FDA-approved panobinostat and of clinically effective adavosertib [32, 61]. Furthermore, notably, our findings also demonstrate that co-treatment with SNDX-5613 and adavosertib exerts superior preclinical in vivo efficacy compared to each drug alone against AML expressing mtNPM1 and FLT3 mutations. Collectively, they strongly support future in vivo testing and development of combination of MI with adavosertib or a pan-HDAC inhibitor in a clinical trial setting in AML with mtNPM1 with or without FLT3 or RAS mutations.

## Supplementary information

Supplemental Figure Legends

Supplemental Materials and Methods

## Data Availability

The RNA-Seq and ChIP-Seq data sets generated and analyzed during the current study are available in the GEO repository as a Super Series under Accession ID # GSE227025.
