# Supplementary material for: Causal linkage of presence of mutant NPM1 to efficacy of novel therapeutic agents against AML cells with mutant NPM1
Source: Leukemia. 2023 Mar 28;37(6):1336–48. doi: 10.1038/s41375-023-01882-4 (PMC10244173; doi:10.1038/s41375-023-01882-4)
Supplement: Supplementary file 1 — Supplemental Figure Legends [file 41375_2023_1882_MOESM1_ESM.pdf]

Supplemental Figure Legends

Figure S1. Knockout of mtNPM1 induces proliferation arrest, depletion of c-Myc via transcription in mtNPM1-expressing AML cells. **A.** Immunoblot analyses of OCI-AML3 cells transfected with sgRNA Ctrl or sgNpm1A and incubated for 8 days **B.** Representative microscopy images of OCI-AML3 cells transfected with sgRNA Ctrl or sgNpm1A and incubated for 7 days. **C-D.** OCI-AML3 cells were transfected with sgRNA Ctrl or sgNpm1A and incubated for 3 days. Cycloheximide (10 μg/mL) was added and incubated for the indicated times. Following this the cells were harvested and immunoblot analyses were conducted on the total cell lysates. Representative blots of three independent experiments are shown for c-Myc and MCL1. c-Myc half-life was quantified in OCI-AML3 sgRNA Ctrl or sgNpm1A. **E.** Onocoplot of NGS-determined mutations in PD AML cells with or without mtNPM1. **F-G.** Percent CD11b or CD14-positive PD wt or mtNPM1 expressing AML cells transfected with sgRNA Ctrl or sgNpm1A and incubated for 12 days.

Figure S2. Depletion of H3K27Ac and H3K4me3 occupancy at the HOX clusters due to mtNPM1A KO in OCI-AML3 cells. A-B. IGV plots of H3K27Ac and H3K4me3 signal density at the HOXA and HOXB loci in OCI-AML3 cells transfected with sgRNA Ctrl or sgNpm1A and incubated for 5 days**.**

Figure S3. Knockout of mtNPM1 attenuates the MEIS1/HOXA9 and E2F transcription factor targets (TFT) in mtNPM1-expressing AML cells. A-B. Gene set enrichment plots of mtNPM1 KO mRNA expressions compared to E2F_Targets (Hallmark) or MEIS1AHOXA9_01 (TFT). **C.** Log2 fold-change in MEIS1/HOXA9 (TFT) genes in OCI-AML3 with mtNPM1 KO over sgRNA Ctrl.

Table S1. LINCS1000-CMap analyses of the mtNPM1 knockout expression signature (RNA-Seq) from OCI-AML3 cells identifies HDAC inhibitors and Wee1 inhibitor as top expression mimickers.

Figure S4. Knockout of mtNPM1 attenuates ATRA, chemotherapy, and AML targeted therapies-induced cell death in OCI-AML3 cells. A-B. Percent apoptotic cells following KO of mtNPM1 and treatment with cytarabine or daunorubicin at the indicated concentrations for 48 hours. **C.** OCI-AML2 cells with and without knock-in of mtNPM1 were cytospun onto glass slides, fixed with paraformaldehyde, permeabilized with Triton X-100, and stained with anti-Npm1, Vimentin, and Fibrillarin antibodies. DAPI was used to stain nuclei. Cells were imaged by spinning-disk confocal microscopy. **D.** Percent apoptotic cells in OCI-AML2 cells with and without CRISPR-mediated knock-in of mtNPM1 treated with cytarabine at the indicated concentrations for 48 hours. * indicates $p < 0.05$ and *** indicates $p < 0.005$ compared to wildtype. **E.** Total bioluminescent flux (p/s) in NSG mice engrafted with luciferase-expressing OCI-AML2 $Npm1^{wt/wt}$ cells and treated for three weeks as indicated. **F.** Kaplan-Meier survival curve of NSG mice engrafted with OCI-AML2 $Npm1^{wt/wt}$ cells and treated with SNDX-5613 as indicated for three weeks. **G.** Percent apoptotic cells following KO of mtNPM1 and treatment with entinostat at the indicated concentrations for 48 hours. **H**. Percent apoptotic cells five days post KO of Wee1 by two guide RNAs compared to sgRNA control. *** indicates $p < 0.005$ and **** indicates $p < 0.001$ compared to sgRNA control.

Figure S5. Co-treatment with SNDX-50469 and Adavosertib or Panobinostat is relatively sparing of normal CD34+ HPCs. A-B. Normal CD34+ progenitor cells (N = 3) were treated with the indicated concentrations of SNDX-50469 and/or Panobinostat or Adavosertib for 96 hours. Percent non-viable cells were determined by flow cytometry.

Table S2. In vitro sensitivity of PD mtNPM1-expressing AML to SNDX-50469 treatment

Table S3. **Co-mutations identified in mtNPM1 + FLT3-ITD AML PDX Luc/GFP**

Suppl. Fig: 1

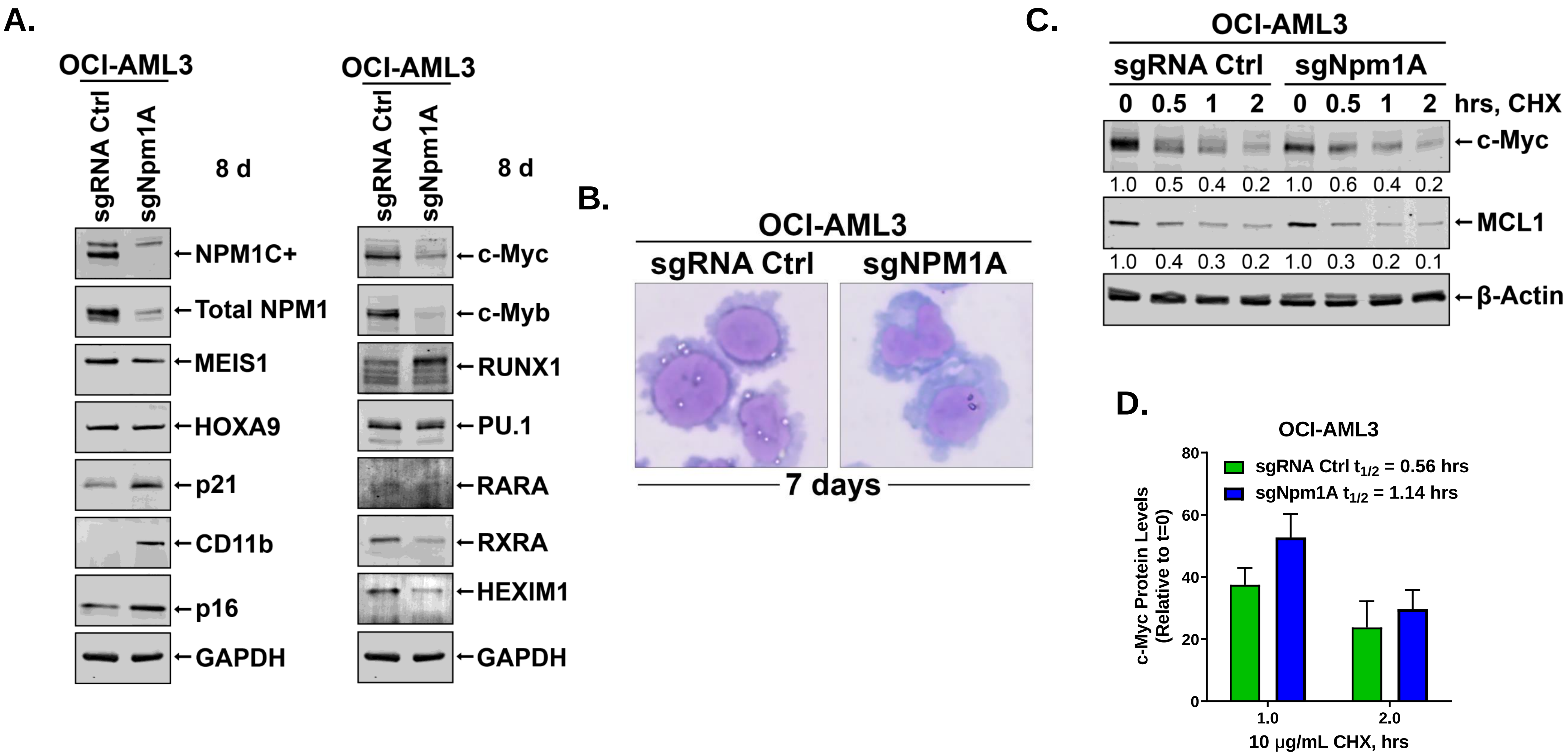
A.
OCI-AML3
sgRNA Ctrl
sgNpm1A
8 d
NPM1C+
Total NPM1
MEIS1
HOXA9
p21
CD11b
p16
GAPDH
OCI-AML3
sgRNA Ctrl
sgNpm1A
8 d
c-Myc
c-Myb
RUNX1
PU.1
RARA
RXRA
HEXIM1
GAPDH
B.
OCI-AML3
sgRNA Ctrl
sgNPM1A
7 days
C.
OCI-AML3
sgRNA Ctrl
sgNpm1A
0 0.5 1 2 0 0.5 1 2 hrs, CHX
c-Myc
1.0 0.5 0.4 0.2 1.0 0.6 0.4 0.2
MCL1
1.0 0.4 0.3 0.2 1.0 0.3 0.2 0.1
β-Actin
D.
OCI-AML3
sgRNA Ctrl t1/2 = 0.56 hrs
sgNpm1A t1/2 = 1.14 hrs
c-Myc Protein Levels
(Relative to t=0)
0
20
40
60
80
1.0
2.0
10 μg/mL CHX, hrs

Suppl. Fig: 1

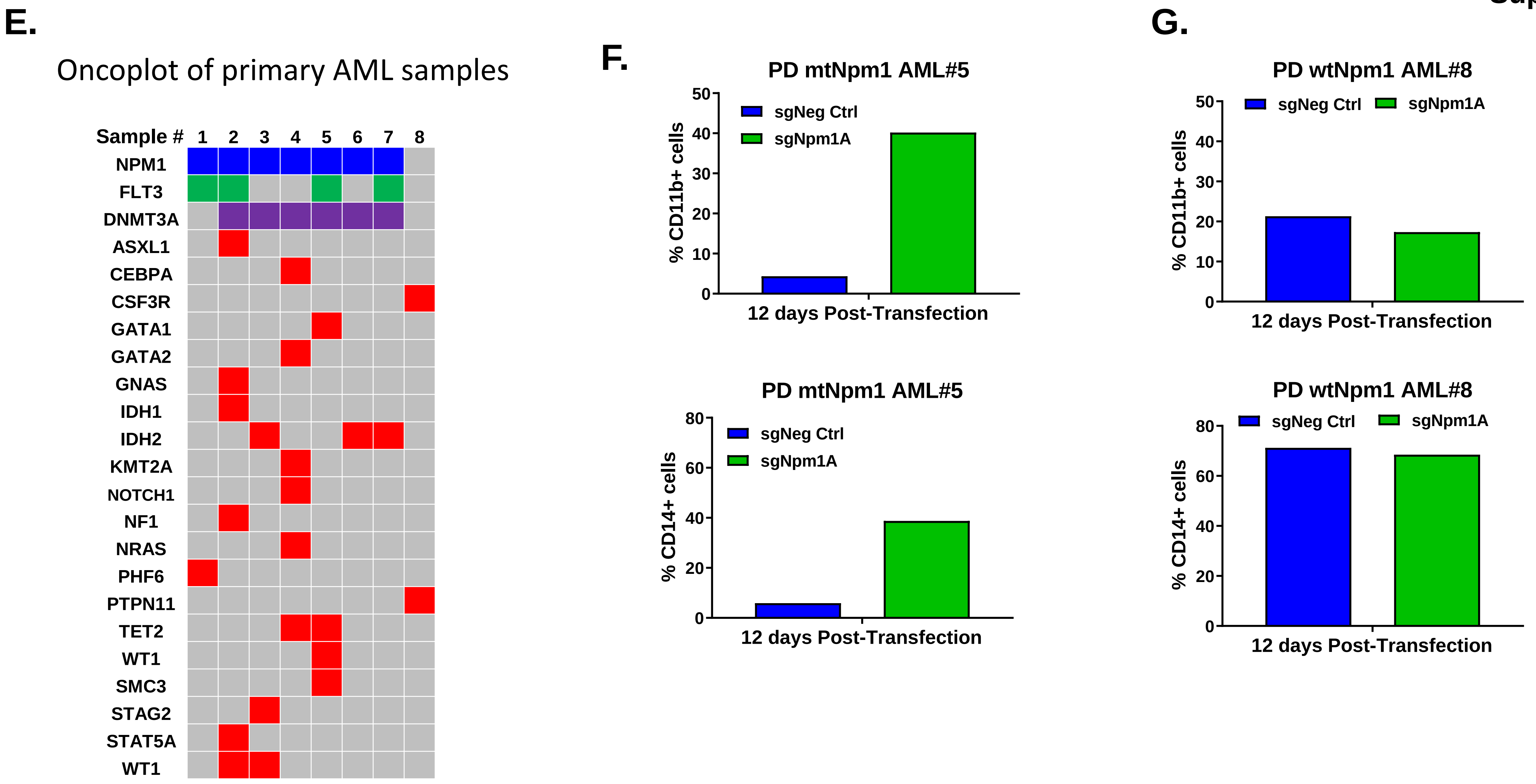

E.
Oncoplot of primary AML samples
Sample #
1
2
3
4
5
6
7
8
NPM1
FLT3
DNMT3A
ASXL1
CEBPA
CSF3R
GATA1
GATA2
GNAS
IDH1
IDH2
KMT2A
NOTCH1
NF1
NRAS
PHF6
PTPN11
TET2
WT1
SMC3
STAG2
STAT5A
WT1
F.
PD mtNpm1 AML#5
% CD11b+ cells
sgNeg Ctrl
sgNpm1A
12 days Post-Transfection
PD mtNpm1 AML#5
% CD14+ cells
sgNeg Ctrl
sgNpm1A
12 days Post-Transfection
G.
PD wtNpm1 AML#8
% CD11b+ cells
sgNeg Ctrl
sgNpm1A
12 days Post-Transfection
PD wtNpm1 AML#8
% CD14+ cells
sgNeg Ctrl
sgNpm1A
12 days Post-Transfection

Suppl. Fig: 2

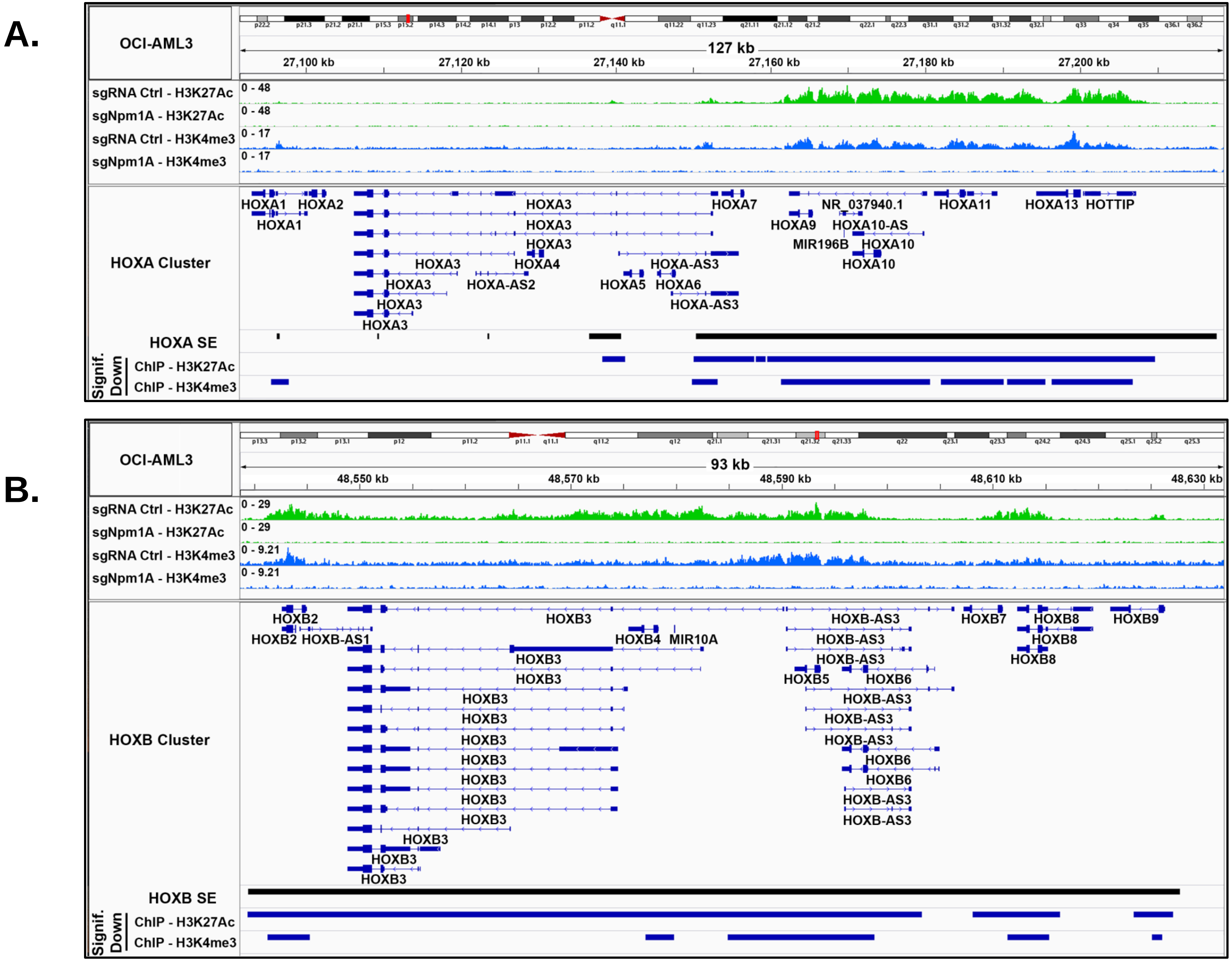
A.
OCI-AML3
127 kb
27,100 kb
27,120 kb
27,140 kb
27,160 kb
27,180 kb
27,200 kb
sgRNA Ctrl - H3K27Ac
0 - 48
sgNpm1A - H3K27Ac
0 - 48
sgRNA Ctrl - H3K4me3
0 - 17
sgNpm1A - H3K4me3
0 - 17
HOXA Cluster
HOXA1
HOXA2
HOXA1
HOXA3
HOXA7
NR_037940.1
HOXA11
HOXA13
HOTTIP
HOXA3
HOXA9
HOXA10-AS
HOXA3
MIR196B
HOXA10
HOXA3
HOXA4
HOXA-AS3
HOXA10
HOXA3
HOXA-AS2
HOXA5
HOXA6
HOXA3
HOXA-AS3
HOXA3
HOXA SE
Signif. Down
ChIP - H3K27Ac
ChIP - H3K4me3
B.
OCI-AML3
93 kb
48,550 kb
48,570 kb
48,590 kb
48,610 kb
48,630 kb
sgRNA Ctrl - H3K27Ac
0 - 29
sgNpm1A - H3K27Ac
0 - 29
sgRNA Ctrl - H3K4me3
0 - 9.21
sgNpm1A - H3K4me3
0 - 9.21
HOXB Cluster
HOXB2
HOXB3
HOXB-AS3
HOXB7
HOXB8
HOXB9
HOXB2
HOXB-AS1
HOXB4
MIR10A
HOXB-AS3
HOXB8
HOXB3
HOXB-AS3
HOXB8
HOXB3
HOXB5
HOXB6
HOXB3
HOXB-AS3
HOXB3
HOXB-AS3
HOXB3
HOXB-AS3
HOXB3
HOXB6
HOXB3
HOXB6
HOXB3
HOXB-AS3
HOXB3
HOXB-AS3
HOXB3
HOXB3
HOXB3
HOXB SE
Signif. Down
ChIP - H3K27Ac
ChIP - H3K4me3

Suppl. Fig: 3

A.

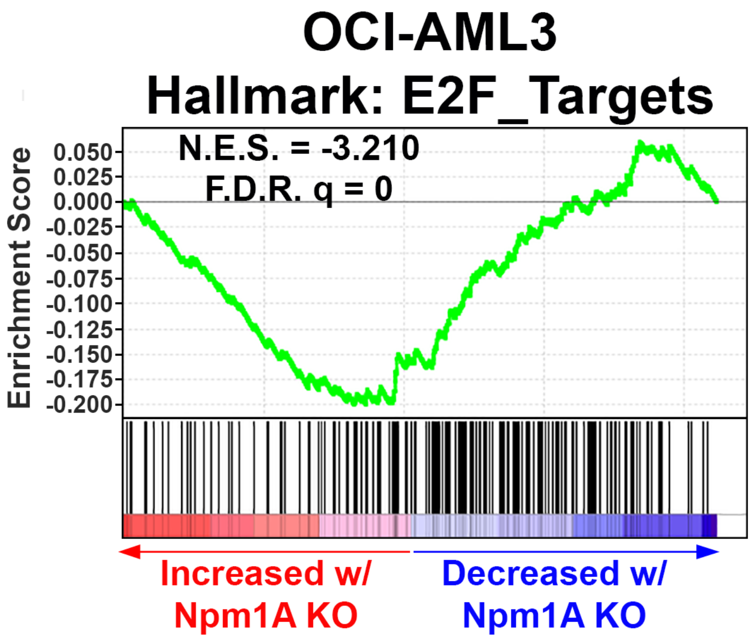

B.

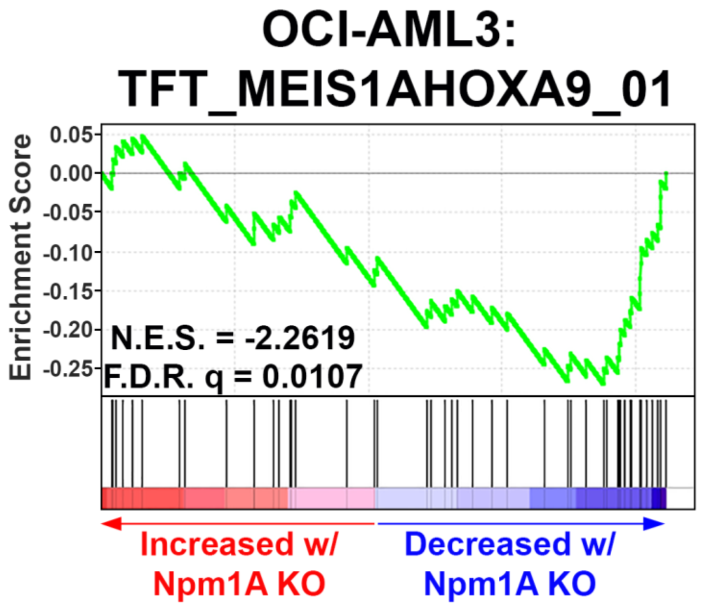

C.

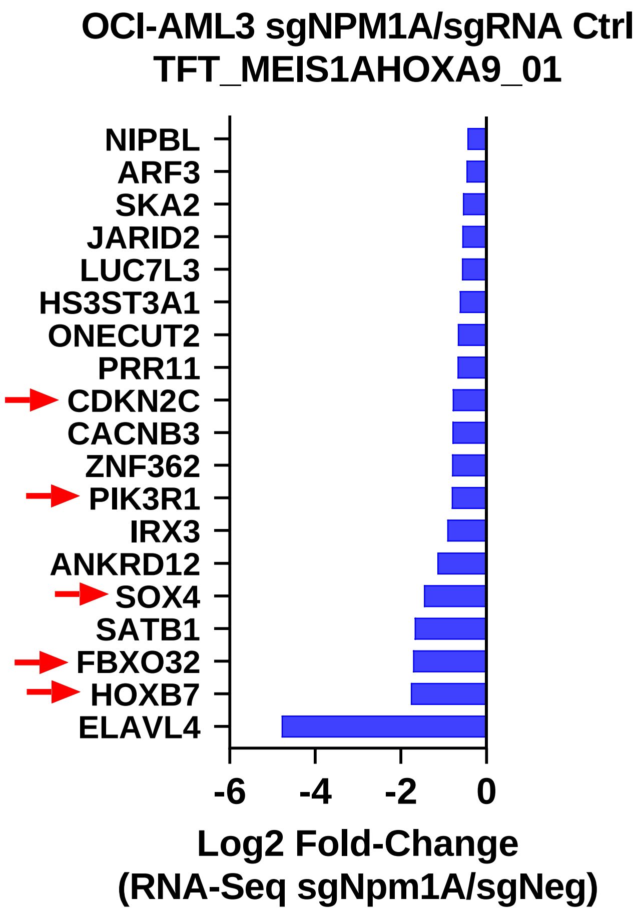

Suppl. Table: 1

LINCS1000

name	description	Rank	target
THM-I-94	HDAC inhibitor, apoptosis stimulant, cell cycle inhibitor	1	HDAC1, HDAC10, HDAC2, HDAC3, HDAC6, HDAC8
halcinonide	glucocorticoid receptor agonist	2	NR3C1
SB-218078	CHK inhibitor, PKC inhibitor	3	CHEK1
trichostatin-a	HDAC inhibitor, CDK expression enhancer, ID1 expression inhibitor	4	HDAC7, HDAC8, HDAC1, HDAC10, HDAC2, HDAC3, HDAC4, HDAC5, HDAC6, HDAC9
kenpaullone	CDK inhibitor, glycogen synthase kinase inhibitor, src inhibitor	5	GSK3B, CDK1, CDK5, CCNB1, CDK2, LCK
L-690488	nositol monophosphatase inhibitor	6	IMPA1
WT-171	HDAC inhibitor	7	HDAC6
vorinostat	HDAC inhibitor, cell cycle inhibitor	8	HDAC1, HDAC2, HDAC3, HDAC6, HDAC8, HDAC10, HDAC11, HDAC5, HDAC9
lasalocid	ionophore antibiotic	9	
MK-1775	wee1 kinase inhibitor (Adavosertib)	10	WEE1
triamcinolone	antiinflammatory agent, corticosteroid agonist, corticosteroid binding globulin binder, corticosteroid hormone receptor agonist, cytochrome P450 inhibitor, glucocorticoid receptor agonist, immunosuppressant, immunosuppressive agent	11	NR3C1, CYP3A5, CYP3A7, SERPINA6
sarmentogenin	ATPase inhibitor	12	ATP1A1
HC-toxin	HDAC inhibitor	13	HDAC1
dacinostat	HDAC inhibitor	14	HDAC1, HDAC2, HDAC3, HDAC4, HDAC5, HDAC6, HDAC7, HDAC8, HDAC9
apicidin	HDAC inhibitor	15	HDAC1, HDAC10, HDAC11, HDAC2, HDAC3, HDAC4, HDAC5, HDAC6, HDAC7, HDAC8, HDAC9
fluocinonide	corticosteroid agonist, corticosteroid hormone receptor agonist	16	NR3C1, SERPINA6, SMO
emetine	protein synthesis inhibitor	17	RPS2
teniposide	topoisomerase inhibitor, DNA inhibitor, DNA repair enzyme inhibitor, mitotic inhibitor	18	TOP2A, CYP3A5
SA-792987	PKC inhibitor	19	WEE1
triptolide	RNA polymerase inhibitor	20	CYP2C19, RELA
fluocinolone	corticosteroid agonist, glucocorticoid receptor agonist	21	NR3C1, SERPINA6
budesonide	glucocorticoid receptor agonist, glucocordicoid receptor antagonist, immunosuppressant	22	NR3C1, CYP3A5, CYP3A7
daunorubicin	RNA synthesis inhibitor, topoisomerase inhibitor, DNA synthesis inhibitor, radical formation stimulant	23	TOP2A, TOP2B
Panobinostat	HDAC inhibitor, apoptosis stimulant, cell cycle inhibitor	24	HDAC1, HDAC2, HDAC3, HDAC4, HDAC6, HDAC7, HDAC8, HDAC9
enzastaurin	PKC inhibitor, AKT inhibitor, angiogenesis inhibitor, apoptosis stimulant, PI3K inhibitor	25	PRKCB, AKT1, GSK3B, PRKCA, PRKCD, PRKCG

Suppl. Fig: 4

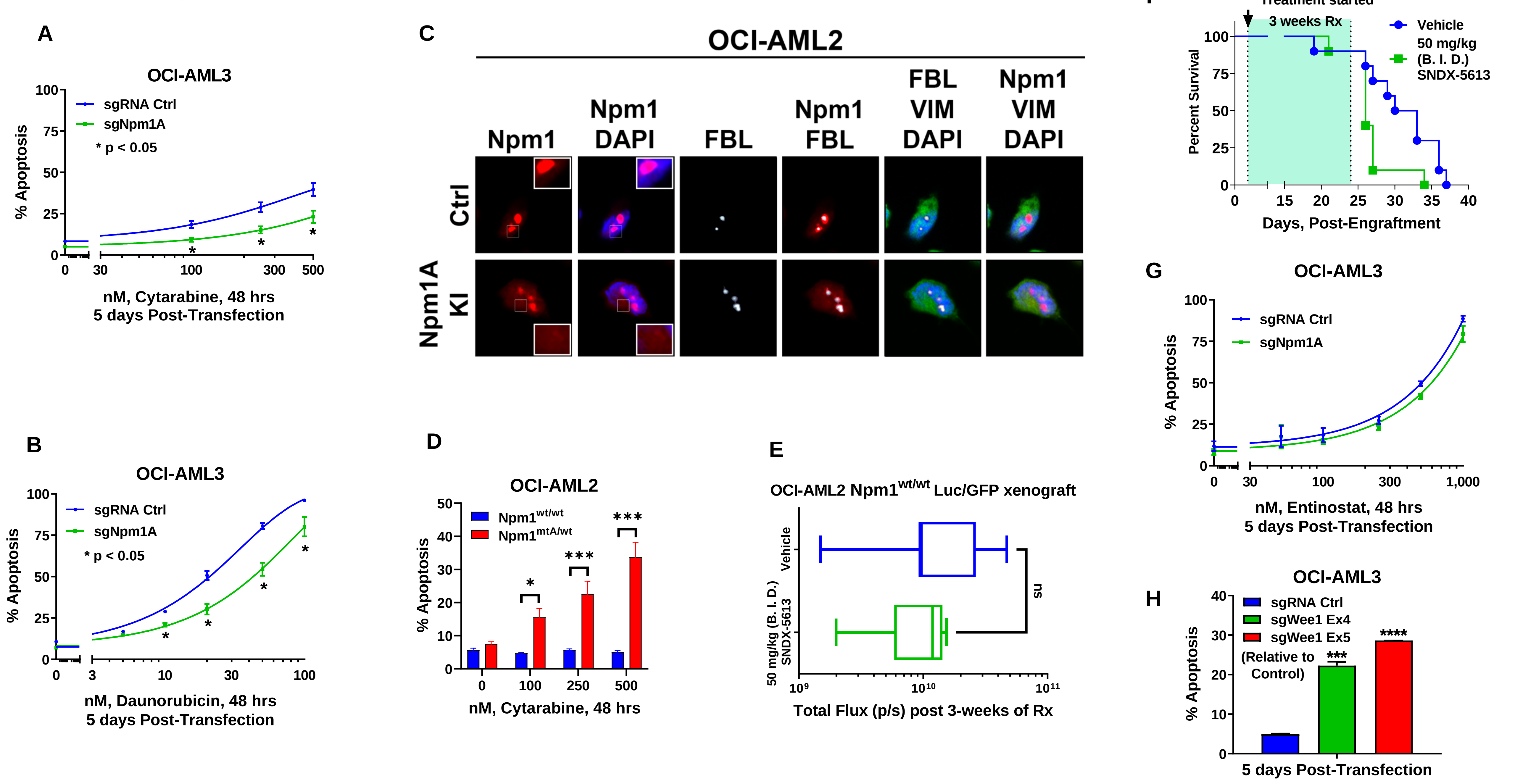
A
OCI-AML3
sgRNA Ctrl
sgNpm1A
* p < 0.05
% Apoptosis
nM, Cytarabine, 48 hrs
5 days Post-Transfection
B
OCI-AML3
sgRNA Ctrl
sgNpm1A
* p < 0.05
% Apoptosis
nM, Daunorubicin, 48 hrs
5 days Post-Transfection
C
OCI-AML2
Npm1
Npm1 DAPI
FBL
Npm1 FBL
FBL VIM DAPI
Npm1 VIM DAPI
Ctrl
Npm1A KI
D
OCI-AML2
Npm1wt/wt
Npm1mtA/wt
% Apoptosis
nM, Cytarabine, 48 hrs
E
OCI-AML2 Npm1wt/wt Luc/GFP xenograft
Vehicle
50 mg/kg (B. I. D.) SNDX-5613
ns
Total Flux (p/s) post 3-weeks of Rx
F
OCI-AML2 Npm1wt/wt Luc/GFP xenograft
Treatment started
3 weeks Rx
Vehicle
50 mg/kg (B. I. D.) SNDX-5613
Percent Survival
Days, Post-Engraftment
G
OCI-AML3
sgRNA Ctrl
sgNpm1A
% Apoptosis
nM, Entinostat, 48 hrs
5 days Post-Transfection
H
OCI-AML3
sgRNA Ctrl
sgWee1 Ex4
sgWee1 Ex5
(Relative to Control)
% Apoptosis
5 days Post-Transfection

Suppl. Fig: 5

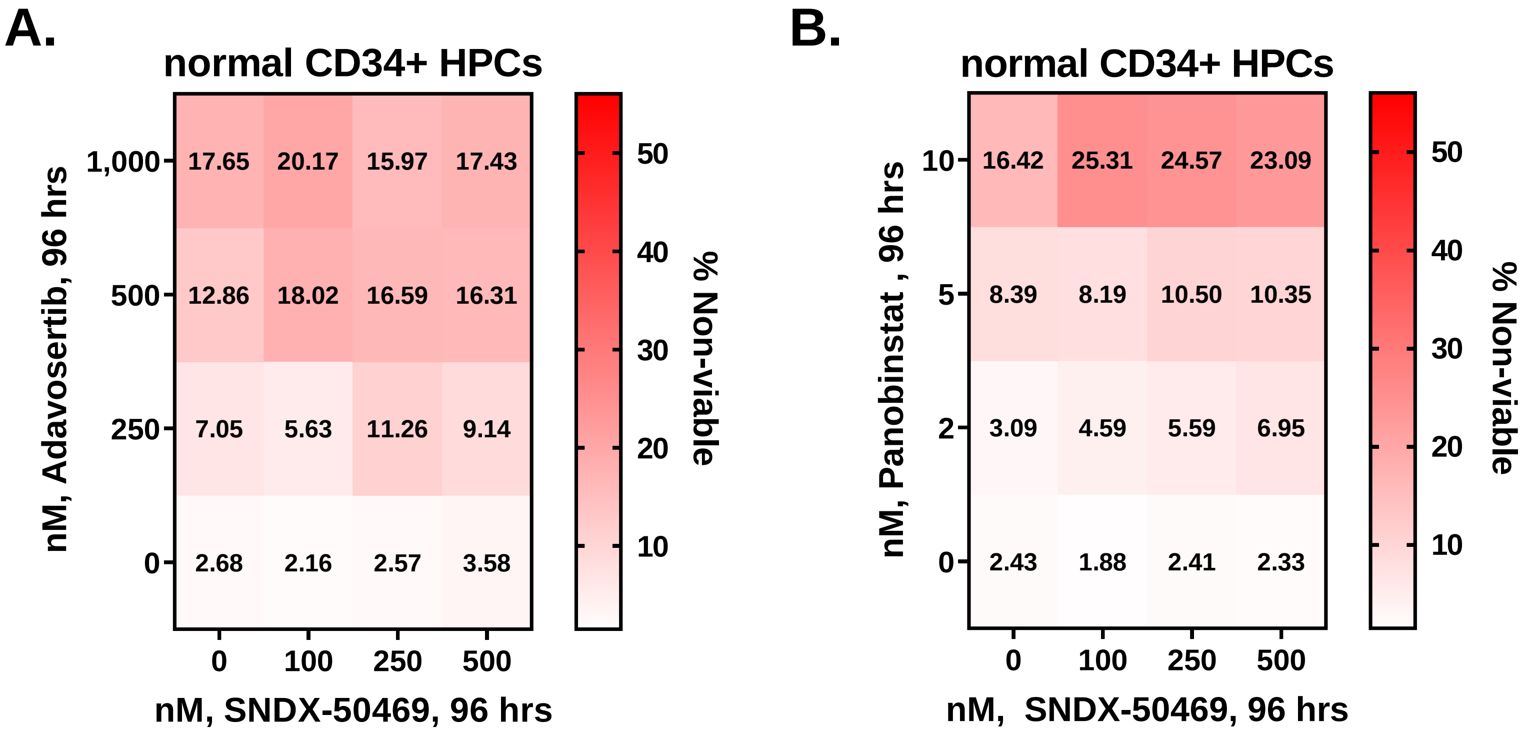
A.
normal CD34+ HPCs
nM, Adavosertib, 96 hrs
1,000
500
250
0
17.65 20.17 15.97 17.43
12.86 18.02 16.59 16.31
7.05 5.63 11.26 9.14
2.68 2.16 2.57 3.58
0 100 250 500
nM, SNDX-50469, 96 hrs
50
40
30
20
10
% Non-viable
B.
normal CD34+ HPCs
nM, Panobinstat , 96 hrs
10
5
2
0
16.42 25.31 24.57 23.09
8.39 8.19 10.50 10.35
3.09 4.59 5.59 6.95
2.43 1.88 2.41 2.33
0 100 250 500
nM, SNDX-50469, 96 hrs
50
40
30
20
10
% Non-viable

Suppl. Table: 2

In vitro sensitivity of PD mtNPM1 expressing AML to SNDX-50469

PD mtNPM1 #	SNDX-50469 (LD_{50})
1	> 10 µM
2	> 10 µM
3	> 10 µM
4	> 10 µM
5	> 10 µM

LD_{50} value calculated utilizing CompuSyn

Suppl. Table: 3

Co-mutations identified in mtNPM1 + FLT3-ITD AML PDX Luc/GFP

Gene	Protein Change	Variant Allele Freq (VAF)
FLT3	ITD	0.50
FLT3	D835Y	0.43
NPM1	W288Cfs*12	0.381
PHF6	G275E	0.940594
ATRX	C1576F	0.980583

FLT3-ITD: start position chr13:28608259 (51-bp)
AGCCAGCTACAGATGGTACAGGTGACCGGCT
CCTCAGATAATGAGTACTCC
