# Supplementary material for: Causal linkage of presence of mutant NPM1 to efficacy of novel therapeutic agents against AML cells with mutant NPM1
Source: Leukemia. 2023 Mar 28;37(6):1336–48. doi: 10.1038/s41375-023-01882-4 (PMC10244173; doi:10.1038/s41375-023-01882-4)
Supplement: Supplementary file 2 — Supplemental Materials and Methods [file 41375_2023_1882_MOESM2_ESM.docx]

**Supplemental Methods:**

**Contact for Reagent sharing. Kapil N. Bhalla. Department of Leukemia, MD. Anderson Cancer Center, 1400 Holcombe Blvd, Unit 428, Houston, TX, 77030. kbhalla@mdanderson.org**

**Reagents and antibodies.** SNDX-5613, SNDX-50469, ziftomenib (KO-539), ATRA, SY-1425, Cytarabine, Daunorubicin, Selinexor (KPT-330), Entinostat, Panobinostat, and Adavosertib for in vitro studies were obtained from MedChem Express (Monmouth Junction, NJ). Cycloheximide was obtained from Santa Cruz Biotechnology, Inc. (Dallas, TX). All compounds were prepared as 10 mM stocks in 100% DMSO and frozen at -80°C in 5-10 µL aliquots to allow for single use, thus avoiding multiple freeze-thaw cycles that could result in compound decomposition and loss of activity. For in vivo studies, SNDX-5613 was obtained from Syndax Pharmaceuticals under an MTA and reconstituted per the manufacturer’s instructions. Anti-Bak [RRID: AB_10828597], anti-Bax [RRID: AB_10557411], anti-BIM [RRID: AB_1030947], anti-Cdk4 [RRID: AB_2631166], anti-CEBPA [RRID: AB_11178517], anti-c-Myb [RRID: AB_2716637], anti-HEXIM1 [RRID: AB_2797969], anti-MCL1 [RRID: AB_2799149], anti-Menin [RRID:AB_10858216], anti-p16 [Cat#: 80772s], anti-p21 [RRID: AB_823586], anti-PU.1 [RRID: AB_10693421], anti-RARA [RRID: AB_2253585], anti-pRb [RRID: AB_11178658], anti-Rb [RRID: AB_823629], anti-Runx1 [RRID: AB_10859035], anti-RXRA [RRID:AB_11140620], antibodies were obtained from Cell Signaling Technologies (Beverly, MA). anti-Bcl2 [RRID: AB_626733], anti-CDK6 [RRID: AB_10610066], anti-GAPDH [RRID: AB_627679], anti-Noxa [RRID: AB_784877] and anti-TP53 [RRID: AB_628086] antibodies were obtained from Santa Cruz Biotechnologies (Dallas, TX). anti-HOXA9 [Cat #: ab140631] antibody was obtained from Abcam (Cambridge, MA). Anti-c-Myc [RRID: AB_10983635] and anti-MEIS1 [RRID: AB_10983635] antibodies were obtained from Thermo Fisher Scientific (Waltham, MA). anti-p27 [RRID: AB_397637] antibody was obtained from BD Biosciences (San Jose, CA). anti-NPM1 [RRID: AB_1842749] antibody was obtained from Sigma-Aldrich, Inc. (St. Louis, MO). Polyclonal anti-NPM1A antibody was generated via a 13 amino acid neomorphic peptide in the c-terminal exon of mtNPM1. Rabbit antiserum was produced and affinity purified by Life Technology (Carlsbad, CA).

**Cell lines and cell culture.** OCI-AML3 [DSMZ Cat# ACC 582] and OCI-AML2 [DSMZ Cat# ACC-99, RRID:CVCL_1619] cells were obtained from the DSMZ. HEK-293T cells were obtained from the Characterized Cell Line Core Facility at M.D. Anderson Cancer Center, Houston TX. All experiments with cell lines were performed within 6 months after thawing or obtaining from DSMZ. The cell lines were also authenticated in the Characterized Cell Line Core Facility at M.D. Anderson Cancer Center, Houston TX. OCI-AML3 were cultured in high-glucose-formulated RPM1 1640 with 20% FBS, 1% NEAA, and 1% penicillin/streptomycin. HEK-293T cells were cultured in high-glucose-formulated DMEM media with 10% FBS, 1% NEAA, 1% L-glutamine, and 1% penicillin/streptomycin. Logarithmically growing, mycoplasma-negative cells were utilized for all experiments. Following drug treatments, cells were washed free of the drug(s) prior to the performance of the studies described.

**Generation of OCI-AML2 NPM1A KI Cell Line Model**

The generation of OCI-AML2 NPM1^mtA/wt^ knock-in (TCTG tandem duplication) models was based on the methods described by Lorenzo Brunetti*^1^*. To target exon 11 that contains the residue to be mutated, the CHOP-CHOP prediction algorithm*^2^* was utilized to develop guide RNA. Due to the lower efficiencies, cr-tracrRNA was used over the more commonly utilized sgRNA in order to introduce a break into only one allele. Synthetic crRNA (3 μL of 400 μM) was annealed with tracrRNA (1.5 μL of 400 μL) at a 2:1 ratio in a 10 μL reaction with 5x annealing buffer via manufacturer’s recommended cycling (Synthego, Inc.) in a thermocycler (Bio-Rad T100 thermal cycler). The RNP (ribonucleoprotein complex) for OCI-AML2 cells was generated by incubating 1.6 pmol of recombinant Cas9 and 7.8 pmol of cr-tracr RNA for 30 minutes at room temperature. The RNP and dsDNA donor (350 ng) were combined in buffer R and electroporated into 2.5e5 of either OCI-AML2 cells utilizing the Neon Transfection System (1600 V, 10 ms, 3 pulses). This was repeated twice and pooled into 2 mL of complete media containing no antibiotics and 2 µM of ROCK inhibitor (Y-27632, Selleck Chemicals; Houston, TX) and allowed to recover. The dsDNA donor contained the NPM1A TCTG tandem duplication, a P2A cleavage site, and the Blasticidin resistance gene flanked by two 400bp homology arms (synthesized by Twist Bioscience). Post-transfection (10 days) knock-in cells were selected for 10 days with the appropriate concentration of Blasticidin (OCI-AML2 15 μg/mL). Following selection, the cells were single cell sorted via flow cytometry. Clones were expanded and then screened for the knock-in mutation. Genomic DNA was harvested and exon 11 was PCR amplified with the Sigma Extract-N-Amp Blood PCR kit (XNAB2R, Sigma-Aldrich; St. Louis, MO). The PCR product was analyzed for presence of the mutation via Sanger sequencing (Eurofins Genomics; Louisville, KY).

**Cell Line Authentication**. The cell lines utilized in these studies were authenticated in the Characterized Cell Line Core Facility at M.D. Anderson Cancer Center, Houston TX utilizing STR profiling.

**Primary AML blasts:** Patient-derived AML cells samples were obtained with informed consent as part of a clinical protocol approved by the Institutional Review Board of The University of Texas, M.D. Anderson Cancer Center. Normal hematopoietic progenitor cells (HPCs) were obtained from delinked, de-identified cord blood samples. Mononuclear cells were purified by Ficoll Hypaque (Axis Shield, Oslo, Norway) density centrifugation following the manufacturer’s protocol. Mononuclear cells were washed once with sterile 1X PBS and suspended in complete RPMI media containing 20% FBS and counted to determine the number of cells isolated prior to immuno-magnetic selection. CD34+ AML blast progenitor cells were purified by immuno-magnetic beads conjugated with anti-CD34 antibody following the manufacturer’s protocol (StemCell Technologies, Vancouver, British Columbia) prior to utilization in the cell viability assays, RNA expression, and immunoblot analyses.

**Sequencing of primary de novo AML blast cells:** We performed targeted next-generation sequencing (NGS) of DNA samples from bone marrow or peripheral blood collected from patients at our center with de novo AML*^3^*. Diagnostic bone marrow samples were obtained for mutational analysis. Total genomic DNA was extracted from unenriched peripheral blood (PB) or bone marrow (BM) samples using ReliaPrep genomic DNA isolation kit (Promega Corp, Madison, WI, USA). Briefly, a total of 250 ng DNA was utilized to prepare sequencing libraries using Agilent HaloPlex custom Kit (Agilent Technologies, Santa Clara, CA, USA). The entire coding sequences of 81 leukemia relevant genes were interrogated on a custom-designed next-generation sequencing approach using the Illumina MiSeq platform (Illumina; San Diego, CA, USA). The genomic reference sequence used was genome GRch37/hg19. The following software tools were utilized in the experimental setup and data analysis: Illumina Experiment Manager 1.6.0 (Illumina; San Diego, CA, USA), MiSeq Control Software 2.4 (Illumina; San Diego, CA, USA), Real Time Analysis 1.18.54 (Illumina; San Diego, CA, USA), Sequence Analysis Viewer 1.8.37 (Illumina; San Diego, CA, USA), MiSeq Reporter 2.5.1 (Illumina; San Diego, CA, USA), and SureCall 3.0.1.4 (Agilent Technologies; Santa Clara, CA, USA). A minimum of 80% reads at quality scores of AQ30 or higher were required to pass quality control. The lower limit of detection of this assay (analytical sensitivity) for single nucleotide variations was determined to be 5% (one mutant allele in the background of nineteen wild type alleles) to 10% (one mutant allele in the background of nine wild type alleles). Testing of patients with active hematologic malignancies was limited to somatic mutations only.

**Analysis of epigenetic state in AML cells *in vitro***. We determined the H3K27Ac [RRID:AB_2793305] and H3K4me3 [RRID:AB_2793611] status in OCI-AML3 cells transfected with sgRNA Ctrl or sgNPM1A and incubated for 5 days by ChIPmentation following a previously described protocol*^4^*, with modifications on the concentration of AmpPure XP beads utilized for dual AmpPure XP SPRI bead selection of the final libraries. We utilized 0.65X beads for the first selection, then a 1.0X bead concentration to narrow the fragment size of the final tagmented ChIP DNA library. ChIP input DNA libraries were only selected with a 1.0X bead concentration. The individual libraries (ChIP and input) were quantified and quality-checked with Qubit and Bioanalyzer 2100 analysis, respectively. The libraries were pooled into one tube, purified utilizing a Qiagen MinElute column, and eluted in 20 µL for loading onto a NextSeq500 sequencer utilizing a mid-output kit. Raw sequence data were mapped to UCSC hg38 (NCBI 51) and log2 fold-changes were calculated with diffReps*^5^* [diffReps, RRID:SCR_010873]. Sequence tracks were visualized with IGV software*^6, 7^* [RRID:SCR_011793]. To identify super enhancers, we performed a ranked order of super enhancers (ROSE) analysis [ROSE, RRID:SCR_017390] utilizing the H3K27Ac status of the chromatin according to the methods of Loven et al.*^8^*. Analysis of transcription factor binding motifs in gained ATAC-Seq peaks was performed with HOMER [HOMER, RRID:SCR_010881].

**Core transcriptional regulatory circuitry (CRC).** CRC profiles for OCI-AML3 sgRNA Ctrl and NPM1A KO were generated with H3K27Ac ChIP-seq data utilizing the CRCmapper algorithm.*^9, 10^* The output result of CRCmapper contained all possible CRCs, ranked based on the average frequency of occurrence of the TFs they contain across all the possible interconnected auto regulatory loops. For each candidate regulatory circuitry, the associated score is calculated by dividing the overall times of occurrence of core TFs across all possible circuitries by the number of core TFs in this circuitry. The top model which contained TFs with the highest frequency of occurrence across all possible circuitries (top scoring circuitry) was selected as the model of CRC in sgRNA Ctrl and sgNPM1A, respectively.

**Transcriptome Analysis.** Total RNA was isolated from OCI-AML3 cells transfected with sgRNA Ctrl or sgNPM1A and incubated for 3 days utilizing a PureLink RNA Mini kit from Ambion, Inc. (Austin, TX). Sequencing libraries were prepared with ERCC spike-in controls in the MD Anderson Cancer Center DNA Sequencing and Microarray core facility and sequenced on an Illumina HiSeq-4000 next generation sequencer [Illumina HiSeq 3000/HiSeq 4000 System, RRID:SCR_016386]. Each library yielded 30-40 million read pairs. Data was mapped using STAR and Samtools (STAR [RRID:SCR_004463], SAMTOOLS [RRID:SCR_002105])*^11, 12^* onto the human genome build UCSC hg38 (NCBI 51) for human data. Gene expression was assessed using DESeq2*^13^* [DESeq2, RRID:SCR_015687], then variance stabilization and quantile normalization were applied. We considered that significance was achieved for fold changes greater than or equal to 1.25X up or down relative to the untreated or parental cells, and p-values less than 0.05. The final p-values were adjusted using the Benjamini & Hochberg method*^14^*. We inferred enriched pathways using the Gene Set Enrichment (GSEA) method*^15^*, and the gene set collection from the Molecular Signature Database (MSigDB)*^16^* [Molecular Signatures Database, RRID:SCR_016863].

**LINCS analysis.** A ranked list of chemical compounds associated with the NPM1A knockout signature was generated using the Library of Integrated Cellular Signatures (LINCS)/Connectivity Map compendia [RRID:SCR_002639] *^17, 18^*. Specifically, significant gene alterations were first separated into up-regulated and down-regulated gene sets, and LINCS was involved programmatically via their API. A ranked summary of the chemical compounds, over-expression of selected proteins, and suppression of selected genes was generated, and ranked by association with the NPM1A knockout signatures in OCI-AML3 cells.

**Plasmid Generation, Viral Packaging, and Creation of Cell Lines.** Plasmid constructs for the production of lentivirus were transfected with packaging plasmids psPAX2 and pMD2.G into HEK-293T cells utilizing jetPRIME reagent (PolyPlus Transfection, New York, NY). The psPAX2 and pMD2.G packaging plasmids were a gift from Didier Trono (Addgene plasmid #12260 and #12259 [RRID: Addgene_12260; RRID: Addgene_12259]). Media was changed the following day. Viral supernatant was collected 72 hours post transfection and filtered through a 0.45 µm PES membrane. AML cells were seeded at 5 x 10^5^ cells/mL in a 50:50 mix of media and lentiviral supernatant with 8 µg/mL polybrene (Sigma-Aldrich). The following day, the viral supernatant was removed by centrifugation and cells were transduced with fresh viral supernatant for an additional 24 hours. To generate luciferase-expressing PDX DF16835 pHIV-Luc-ZsGreen (a gift from Bryan Welm [Addgene plasmid #39196; http://n2t.net/addgene:39196; RRID: Addgene_39196]) was packaged as above and transduced into PDX DF16835 cells. PDX DF16835 cells were expanded in mice and ZsGreen-positive cells were sorted by flow cytometry (FACSAria, FL-1 channel, top GFP-expressing cells) for their utilization in therapeutic in vivo mouse studies.

**Confocal immunofluorescence microscopy.** sgRNA transfected OCI-AML3 cells or OCI-AML2 with or without mtNPM1 knock-in were cytospun onto glass slides to prepare for confocal microscopy. Cells were fixed with 4% paraformaldehyde in 1X PBS for 10 minutes, then washed three times with 1X PBS. Next, cells were permeabilized with 0.5% Triton X-100 for 5 minutes, then rinsed three times with 1X PBS. Cells were blocked in 3% BSA/PBS + 1% FBS for 1 hour at room temperature. Next, the primary antibody for NPM1 [RRID: AB_1842749] was added and the cells/slides were incubated in a humidified chamber overnight at 4^o^ C. Excess antibody was removed by washing the slides three times with 1X PBS. Anti-mouse Alexa 594-conjugated secondary antibody (Invitrogen, Carlsbad, CA) was added and the slides were incubated in a humidified chamber for 1-2 hours in the dark. Excess antibody was removed by washing three times with 1X PBS. Then the slides was incubated with the primary antibodies AlexaFluor 647 conjugated to Fibrillarin [sc-166021; RRID:AB_2105797] or AlexaFluor 488 conjugated Vimentin [sc-6260; RRID: AB_2199796] in a humidified chamber overnight at 4^o^ C. Excess antibody was removed by washing three times with 1X PBS. Nuclei were counterstained with DAPI (#62248, ThermoFisher), and then coverslips were mounted onto the slides utilizing Prolong Diamond anti-fade mountant (P36970, LifeTech, Carlsbad, CA). Imaging was performed on a Andor Revolution XDi WD spinning disk confocal microscope in the MD Anderson Flow Cytometry and Imaging Core. Images were obtained with a 60X oil immersion objective. Confocal analysis was performed at least twice. Representative images are shown for each condition.

**Cell cycle analysis of AML cells**. OCI-AML3 cells transfected with sgRNA Ctrl or sgNPM1A and incubated for 5 days were harvested by centrifuging at 125 x g for 5 minutes. Cells were washed twice with 1× phosphate-buffered saline (PBS) in 12 x 75 mm flow tubes, re-suspended in 200 µL of 1X PBS and fixed in 70% ethanol by adding 800 µL of molecular grade 70% ethanol dropwise to the cells in the tube. The tubes were then vortexed to mix and stored overnight at -20°C. Fixed cells were washed twice with 1× PBS by centrifuging at 125 x g for 5 minutes and then stained in 250 µL of DNA staining buffer [5 mL Triton-PBS (100 µL of Triton X100 in 100 mL of 1X PBS) with 100 µL of 1 mg/mL propidium iodide and 100 µL of 10mg/mL RNAse A] in the dark for 15 minutes at 37°C. Cell-cycle data were collected on a flow cytometer with a 488 nM laser in the FL-2 channel and analyzed with Accuri CFlow6 software (BD Biosciences).

**Assessment of apoptosis by annexin-V staining.** Untreated or drug-treated control, Npm1A knockout, or Wee1 knockout cells were stained with Annexin-V (Pharmingen, San Diego, CA) and TO-PRO-3 iodide (Life Technologies, Carlsbad, CA) and the percentages of apoptotic cells were determined by flow cytometry after 48 hours.

**Assessment of percentage non-viable cells.** Following designated treatments, OCI-AML3, OCI-AML2 cells or PD AML cells were stained with propidium iodide or TO-PRO-3 iodide (Life Technologies, Carlsbad, CA) and analyzed by flow cytometry on a BD Accuri CFlow-6 flow cytometer (BD Biosciences, San Jose, CA). To analyze synergism, cells were treated with combinations for 96 hours and the percentages of TO-PRO-3 iodide-positive, non-viable cells were determined by flow cytometry. We utilized matrix dosing of agents in combinations to allow synergy assessment by ZIP scoring utilizing the SynergyFinderV3 online web application tool (<http://synergyfinder.fimm.fi/>)*^19-21^*.

**Assessment of leukemia cell differentiation.** Untreated or treated (DMSO) OCI-AML3 (sgRNA Ctrl or sgNPM1A) or PD AML cells were harvested and washed with 1X PBS. Cells were re-suspended in 0.5% BSA/PBS and stained with PE-conjugated anti-CD14 [RRID:AB_395799] or APC-conjugated anti-CD11b antibody [RRID:AB_398456] or PE-conjugated IgG1 isotype control [RRID:AB_395953], and APC-conjugated IgG1 isotype control antibody [RRID:AB_398613] in the dark, on ice for 30 minutes. Cells were washed with 0.5% BSA/PBS by centrifugation at 125 x g for 5 minutes, and then re-suspended in 0.5% BSA/PBS for analysis by flow cytometry. Cells were assessed in the FL-2 and FL-4 fluorescence channels. Differentiation of leukemia cells was also determined by examination of cellular/nuclear morphology. Cells were cytospun onto glass slides at 500 rpm for 5 minutes. The cytospun cells were fixed and stained with a Protocol® HEMA3 stain set (Fisher Scientific, Kalamazoo, MI). Cellular/nuclear morphology was assessed by light microscopy. Two hundred cells were counted in at least five sections of the slide for each condition. The % morphologic differentiation is reported relative to control cells.

**RNA isolation and quantitative polymerase chain reaction.** Following the designated treatments, total RNA was isolated from sAML cells utilizing a PureLink RNA Mini kit from Ambion, Inc. (Austin, TX) and reverse transcribed with a High Capacity Reverse Transcription kit from Life Technologies (Carlsbad, CA). Quantitative real-time PCR analysis for the expression of target genes was performed on cDNA using TaqMan probes and a TaqMan Universal PCR Mastermix from Applied Biosystems (Foster City, CA). Relative mRNA expression was normalized to the expression of GAPDH and compared to the untreated cells. Additionally, we probed upstream of protein synthesis by utilizing a TaqMan array (4414196, ThermoFisher Scientific) in order to determine changes in the levels of tRNA-associated genes.

**Cell lysis and protein quantitation.** Knockout**,** untreated, or drug-treated cells were centrifuged, and the cell pellets were incubated in lysis buffer on ice for 20 minutes*^22^*. After centrifugation, an aliquot of each cell lysate was diluted 1:10 and the protein content was quantitated using a BCA protein quantitation kit (Pierce, Rockford, IL), according to the manufacturer’s protocol. Protein concentrations were determined by comparing the absorbance at 562 nm compared to a known concentration range of bovine serum albumin (BSA) from 0.125 mg to 2 mg/mL.

**SDS-PAGE and immunoblot analyses.** Thirty micrograms of total cell lysate were used for SDS-PAGE. Western blot analyses were performed on total cell lysates using specific antisera or monoclonal antibodies. Blots were washed with 1× PBST, then incubated in IRDye 680RD goat anti-mouse (RRID:AB_10956588) or IRDye 800CW goat anti-rabbit (RRID:AB_621843) secondary antibodies (LI-COR, Lincoln, NE) for 1 h, washed three times in 1× Phosphate Buffered Saline with Tween®20 (PBST) and scanned with an Odyssey CLX Infrared Imaging System utilizing Image Studio 5.0 Software (RRID:SCR_015795) (LI-COR, Lincoln, NE). The expression levels of β-Actin or GAPDH in the cell lysates were used as the loading control for the western blots. Immunoblot analyses were performed at least twice. Representative immunoblots were subjected to densitometry analysis. Densitometry analysis was performed using ImageJ software*^23^*.

**In vivo model of de novo AML:** All in vivo studies were approved by and conducted in accordance with the guidelines of the IACUC at the M.D. Anderson Cancer Center, an AAALAC-accredited facility. Female NOD.Cg-Prkdc^scid^ Il2rg^tm1Wjl^/SzJ (NSG) mice (stock number: 005557; 4-6 weeks of age) [Jackson Labs, Bar Harbor, ME; RRID: IMSR_JAX:005557] were exposed to 2.5 Gy of radiation. The following day, mice were injected in the lateral tail vein. Mice were imaged utilizing a Xenogen Lumina in vivo imaging system to document engraftment before treatment was initiated. Mice in all models described below were randomized into groups based on equivalent mean bioluminescent intensity to control for variation in cell engraftment and variation between different treatment groups.

For the OCI-AML3 model, mice (n=10 per cohort) were injected in the lateral tail vein with 2.0 x 10^6^ GFP-luciferase expressing OCI-AML3 and monitored for 5 days. Treatment was initiated on day 6. Cohort one mice were treated with 50 mg/kg of SNDX-5613 BID, daily for 3 weeks by oral gavage. In the second cohort mice were treated with 3.5 mg/kg of Panobinostat 3 times per week for 3 weeks by intraperitoneal injection. The third cohort of mice were treated with 60 mg/kg of Adavosertib BID daily for 3 weeks by oral gavage. Mice were imaged weekly by bioluminescent imaging to document treatment efficacy and/or disease progression. Total bioluminescence was recorded as photons/second. Mice that became moribund or experienced hind limb paralysis were euthanized according to the approved IACUC protocol. Department of Veterinary Medicine staff members assisting in determining when euthanasia was required were blinded to the experimental conditions of the study. The survival of the mice is represented by a Kaplan-Meier plot. Significance was determined by a Mantel-Cox log rank test. P-values of less than 0.05 were assigned significance.

For the OCI-AML2 models, mice (n=10 per cohort) were injected in the lateral tail vein with 1.0 x 10^6^ GFP-luciferase expressing OCI-AML2 NPM1^wt/wt^ or 3.0 x 10^6^ GFP-luciferase expressing OCI-AML2 NPM1^mtA/wt^ and monitored for 5 days. Treatment was initiated on day 6. The cohorts were treated with 50 mg/kg of SNDX-5613 BID, daily for 3 or 10 weeks as indicated by oral gavage. Mice were imaged weekly by bioluminescent imaging to document treatment efficacy and/or disease progression. Total bioluminescence was recorded as photons/second. Mice that became moribund or experienced hind limb paralysis were euthanized according to the approved IACUC protocol. Department of Veterinary Medicine staff members assisting in determining when euthanasia was required were blinded to the experimental conditions of the study. The survival of the mice is represented by a Kaplan-Meier plot. Significance was determined by a Mantel-Cox log rank test. P-values of less than 0.05 were assigned significance.

For the DF16835 mouse model, mice (n=10 per cohort) were injected in the lateral tail vein with 7.5 x 10^5^ GFP-luciferase expressing DF16835 mtNPM1, FLT3-ITD AML PDX (from Dana Farber) and monitored for 11 days. Treatment was initiated on day 12. The first cohort of mice were treated with 3.5 mg/kg of Panobinostat 3 times per week for 2 weeks, followed by a week at 3.5 mg/kg and then 3 additional weeks at 2.5 mg/kg by intraperitoneal injection. The second cohort of mice were treated with 60 mg/kg of Adavosertib BID daily for 6 weeks by oral gavage. All mice in each treatment cohort were imaged utilizing a Xenogen Lumina in vivo imaging system once per week to monitor disease status and treatment efficacy. Total bioluminescence was recorded as photons/second. Mice that became moribund or experienced hind limb paralysis were euthanized according to the approved IACUC protocol. Department of Veterinary Medicine staff members assisting in determining when euthanasia was required were blinded to the experimental conditions of the study. The survival of the mice is represented by a Kaplan-Meier plot. Significance was determined by a Mantel-Cox log rank test. P-values of less than 0.05 were assigned significance.

For the AML#5 mouse model, mice (n=10 per cohort) were injected in the lateral tail vein with 2 x 10^6^ GFP-luciferase expressing mtNPM1, FLT3-ITD, FLT3-F691L AML PDX and monitored for 5 days. Treatment was initiated on day 6. The first cohort of mice were treated with 60 mg/kg of Adavosertib BID daily by oral gavage for 2 weeks and then reduced to 40 mg/kg for 2 weeks. The second cohort of mice were treated with 50 mg/kg of SNDX-5613 BID daily by oral gavage for 2 weeks and then reduced to 33.3 mg/kg for 2 weeks. For the final cohort, mice were treated with SNDX-5613 and Adavosertib at the dose and schedule indicated above. All mice in each treatment cohort were imaged utilizing a Xenogen Lumina in vivo imaging system once per week to monitor disease status and treatment efficacy. Total bioluminescence was recorded as photons/second. The survival of the mice is represented by a Kaplan-Meier plot. Significance was determined by a Mantel-Cox log rank test. P-values of less than 0.05 were assigned significance.

**Power analysis for in vivo studies**. With a sample size of 10 mice per group, we can achieve 79.5% power to detect a difference of overall survival at a significance level of 0.05 with one-sided log-rank test, assuming 30% of mouse-survival at the end of study in the experimental group.

**Statistical analysis**. Significant differences between values obtained in AML cells treated with different experimental conditions compared to untreated control cells were determined using the Student’s t-test in GraphPad V8. For the *in vivo* mouse models, a two-tailed, unpaired t-test was utilized for comparing total bioluminescent flux. For survival analysis, a Kaplan-Meier plot and a Mantel–Cox log rank test were utilized for comparisons of different cohorts. P values of < 0.05 were assigned significance.

**Data and Software availability**. RNA-Seq and ChIP-Seq datasets have been deposited as a Series in GEO and assigned accession ID # GSE227025.

**REFERENCES for Supplemental Methods**

[1] Brunetti, L., Gundry, M. C., Sorcini, D., Guzman, A. G., Huang, Y. H., Ramabadran, R., Gionfriddo, I., Mezzasoma, F., Milano, F., Nabet, B., Buckley, D. L., Kornblau, S. M., Lin, C. Y., Sportoletti, P., Martelli, M. P., Falini, B., and Goodell, M. A. (2018) Mutant NPM1 Maintains the Leukemic State through HOX Expression, *Cancer Cell* *34*, 499-512 e499.

[2] Labun, K., Montague, T. G., Krause, M., Torres Cleuren, Y. N., Tjeldnes, H., and Valen, E. (2019) CHOPCHOP v3: expanding the CRISPR web toolbox beyond genome editing, *Nucleic Acids Res* *47*, W171-W174.

[3] Khan, M., Cortes, J., Kadia, T., Naqvi, K., Brandt, M., Pierce, S., Patel, K. P., Borthakur, G., Ravandi, F., Konopleva, M., Kornblau, S., Kantarjian, H., Bhalla, K., and DiNardo, C. D. (2017) Clinical Outcomes and Co-Occurring Mutations in Patients with RUNX1-Mutated Acute Myeloid Leukemia, *Int J Mol Sci* *18*.

[4] Schmidl, C., Rendeiro, A. F., Sheffield, N. C., and Bock, C. (2015) ChIPmentation: fast, robust, low-input ChIP-seq for histones and transcription factors, *Nat Methods* *12*, 963-965.

[5] Shen, L., Shao, N. Y., Liu, X., Maze, I., Feng, J., and Nestler, E. J. (2013) diffReps: detecting differential chromatin modification sites from ChIP-seq data with biological replicates, *PLoS One* *8*, e65598.

[6] Robinson, J. T., Thorvaldsdottir, H., Winckler, W., Guttman, M., Lander, E. S., Getz, G., and Mesirov, J. P. (2011) Integrative genomics viewer, *Nat Biotechnol* *29*, 24-26.

[7] Thorvaldsdottir, H., Robinson, J. T., and Mesirov, J. P. (2013) Integrative Genomics Viewer (IGV): high-performance genomics data visualization and exploration, *Brief Bioinform* *14*, 178-192.

[8] Loven, J., Hoke, H. A., Lin, C. Y., Lau, A., Orlando, D. A., Vakoc, C. R., Bradner, J. E., Lee, T. I., and Young, R. A. (2013) Selective inhibition of tumor oncogenes by disruption of super-enhancers, *Cell* *153*, 320-334.

[9] Huang, M., Chen, Y., Yang, M., Guo, A., Xu, Y., Xu, L., and Koeffler, H. P. (2018) dbCoRC: a database of core transcriptional regulatory circuitries modeled by H3K27ac ChIP-seq signals, *Nucleic Acids Res* *46*, D71-D77.

[10] Saint-Andre, V., Federation, A. J., Lin, C. Y., Abraham, B. J., Reddy, J., Lee, T. I., Bradner, J. E., and Young, R. A. (2016) Models of human core transcriptional regulatory circuitries, *Genome Res* *26*, 385-396.

[11] Dobin, A., Davis, C. A., Schlesinger, F., Drenkow, J., Zaleski, C., Jha, S., Batut, P., Chaisson, M., and Gingeras, T. R. (2013) STAR: ultrafast universal RNA-seq aligner, *Bioinformatics* *29*, 15-21.

[12] Li, H., Handsaker, B., Wysoker, A., Fennell, T., Ruan, J., Homer, N., Marth, G., Abecasis, G., Durbin, R., and Genome Project Data Processing, S. (2009) The Sequence Alignment/Map format and SAMtools, *Bioinformatics* *25*, 2078-2079.

[13] Love, M. I., Huber, W., and Anders, S. (2014) Moderated estimation of fold change and dispersion for RNA-seq data with DESeq2, *Genome Biol* *15*, 550.

[14] Benjamini, Y., and Hochberg, Y. (1995) Controlling the False Discovery Rate: A Practical and Powerful Approach to Multiple Testing, *Journal of the Royal Statistical Society: Series B (Methodological)* *57*, 289-300.

[15] Subramanian, A., Tamayo, P., Mootha, V. K., Mukherjee, S., Ebert, B. L., Gillette, M. A., Paulovich, A., Pomeroy, S. L., Golub, T. R., Lander, E. S., and Mesirov, J. P. (2005) Gene set enrichment analysis: a knowledge-based approach for interpreting genome-wide expression profiles, *Proc Natl Acad Sci U S A* *102*, 15545-15550.

[16] Liberzon, A., Subramanian, A., Pinchback, R., Thorvaldsdottir, H., Tamayo, P., and Mesirov, J. P. (2011) Molecular signatures database (MSigDB) 3.0, *Bioinformatics* *27*, 1739-1740.

[17] Lamb, J., Crawford, E. D., Peck, D., Modell, J. W., Blat, I. C., Wrobel, M. J., Lerner, J., Brunet, J. P., Subramanian, A., Ross, K. N., Reich, M., Hieronymus, H., Wei, G., Armstrong, S. A., Haggarty, S. J., Clemons, P. A., Wei, R., Carr, S. A., Lander, E. S., and Golub, T. R. (2006) The Connectivity Map: using gene-expression signatures to connect small molecules, genes, and disease, *Science* *313*, 1929-1935.

[18] Subramanian, A., Narayan, R., Corsello, S. M., Peck, D. D., Natoli, T. E., Lu, X., Gould, J., Davis, J. F., Tubelli, A. A., Asiedu, J. K., Lahr, D. L., Hirschman, J. E., Liu, Z., Donahue, M., Julian, B., Khan, M., Wadden, D., Smith, I. C., Lam, D., Liberzon, A., Toder, C., Bagul, M., Orzechowski, M., Enache, O. M., Piccioni, F., Johnson, S. A., Lyons, N. J., Berger, A. H., Shamji, A. F., Brooks, A. N., Vrcic, A., Flynn, C., Rosains, J., Takeda, D. Y., Hu, R., Davison, D., Lamb, J., Ardlie, K., Hogstrom, L., Greenside, P., Gray, N. S., Clemons, P. A., Silver, S., Wu, X., Zhao, W. N., Read-Button, W., Wu, X., Haggarty, S. J., Ronco, L. V., Boehm, J. S., Schreiber, S. L., Doench, J. G., Bittker, J. A., Root, D. E., Wong, B., and Golub, T. R. (2017) A Next Generation Connectivity Map: L1000 Platform and the First 1,000,000 Profiles, *Cell* *171*, 1437-1452 e1417.

[19] Ianevski, A., Giri, A. K., and Aittokallio, T. (2020) SynergyFinder 2.0: visual analytics of multi-drug combination synergies, *Nucleic Acids Res* *48*, W488-W493.

[20] Ianevski, A., He, L., Aittokallio, T., and Tang, J. (2020) SynergyFinder: a web application for analyzing drug combination dose-response matrix data, *Bioinformatics* *36*, 2645.

[21] Ianevski, A., Giri, A. K., and Aittokallio, T. (2022) SynergyFinder 3.0: an interactive analysis and consensus interpretation of multi-drug synergies across multiple samples, *Nucleic Acids Res*.

[22] Fiskus, W., Verstovsek, S., Manshouri, T., Rao, R., Balusu, R., Venkannagari, S., Rao, N. N., Ha, K., Smith, J. E., Hembruff, S. L., Abhyankar, S., McGuirk, J., and Bhalla, K. N. (2011) Heat shock protein 90 inhibitor is synergistic with JAK2 inhibitor and overcomes resistance to JAK2-TKI in human myeloproliferative neoplasm cells, *Clin Cancer Res* *17*, 7347-7358.

[23] Schneider, C. A., Rasband, W. S., and Eliceiri, K. W. (2012) NIH Image to ImageJ: 25 years of image analysis, *Nat Methods* *9*, 671-675.
